# Clinical Phenotypes of Oral and Maxillofacial Space Infection: Derivation, External Assessment, and Prognostic Evaluation

**DOI:** 10.3390/jcm15218414

**Published:** 2026-10-29

**Authors:** Baotian Zhang, Kaixiao Yan, Liao Wang, Wei Zeng, Yutao Xiong, Wei Tang, Chang Liu

**Affiliations:** 1 State Key Laboratory of Oral Diseases, National Center for Stomatology, National Clinical Research Center for Oral Diseases, West China Hospital of Stomatology, Sichuan University, Chengdu 610041, China; baotianzhang@foxmail.com (B.Z.); wangliaonew@163.com (L.W.); zengwei_2024@foxmail.com (W.Z.); xiongyutaod@sina.com (Y.X.); 2 Department of Oral and Maxillofacial Surgery, Qilu Hospital of Shandong University, Jinan 250012, China; 3 Center for Oral Health, Ruijin Hospital, Shanghai Jiao Tong University School of Medicine, Shanghai 200025, China; yankaixiao008@gmail.com; 4 Department of Oral and Maxillofacial Surgery, University of Michigan School of Dentistry, Ann Arbor, MI 48109, USA

**Keywords:** oral and maxillofacial space infection, clinical phenotyping, oral and maxillofacial surgery, clinical heterogeneity

## Abstract

**Background/Objectives**: Oral and maxillofacial space infection (OMSI) has heterogeneous clinical presentations. We aimed to derive candidate clinical phenotypes at hospital presentation and assess their external structural reproducibility and prognostic value beyond anatomical severity. **Methods:** This two-center retrospective study included 287 patients in the development cohort and 51 in the external cohort. Consensus k-means clustering was performed using 24 presentation variables across 20 imputed datasets, followed by frozen external phenotype assignment. Firth’s bias-reduced logistic regression was used to fit the traditional severity score (TSS), phenotype-only, and combined models for the recorded primary composite outcome of intensive care unit admission, tracheostomy, septic shock, or descending mediastinal infection. Phenotype assignments remained fixed during bootstrap internal validation. **Results:** Four candidate phenotypes showed different inflammatory, physiological, and metabolic profiles. The febrile and glucose-dominant phenotypes both had elevated inflammatory markers but differed in temperature and glucose levels. A history of diabetes was recorded in 29 of 31 patients in the glucose-dominant phenotype. The primary composite outcome occurred in 35/287 (12.2%) and 22/51 (43.1%) patients in the development and external cohorts, respectively. Independent external clustering showed limited agreement with the frozen assignments (adjusted Rand index = 0.320). Bootstrap optimism-corrected areas under the receiver operating characteristic curve (AUCs) were 0.798, 0.631, and 0.799 for the TSS, phenotype-only, and combined models, respectively. The corresponding external AUCs were 0.926, 0.665, and 0.891. All three frozen models underestimated absolute risk in the external cohort (observed-to-expected ratios: 4.425, 3.620, and 4.424, respectively). **Conclusions:** The candidate phenotypes described overlapping inflammatory, physiological, and metabolic patterns at presentation. Independent external clustering showed limited structural agreement, and adding phenotype membership to TSS did not consistently improve prognostic discrimination. Their role in clinical decision-making requires further evaluation.

## 1. Introduction

Oral and maxillofacial space infection (OMSI) can spread through the fascial spaces of the head and neck, leading to airway compromise, mediastinal infection, or systemic complications [1,2]. The traditional severity score (TSS), developed by Flynn et al., quantifies the anatomical extent of infection [3,4,5] but does not fully capture the diversity of clinical presentations. Previous studies of odontogenic and deep-neck infections have linked anatomical involvement and inflammatory markers to disease severity and clinical outcomes [6,7,8,9]. Clinical phenotyping using routinely available clinical variables has been explored in sepsis to characterize patient heterogeneity [10,11]. A recent systematic review of OMSI identified diabetes, elevated body temperature, multiple-space involvement, and inflammatory markers as factors associated with complications [12]. A prospective multicenter study of cervicofacial infections also identified airway-related signs, inflammatory measurements, and anatomical involvement as factors associated with critical-care admission [13].

Unsupervised phenotyping may help characterize how routinely recorded clinical variables occur together at hospital presentation. However, studies assessing the cross-center reproducibility of OMSI phenotypes and their prognostic contribution beyond TSS remain limited.

We conducted an exploratory two-center study to derive candidate clinical phenotypes of OMSI from routinely available clinical data at hospital presentation, assess their reproducibility in an external cohort, and examine their associations with recorded clinical outcomes. We also compared the TSS, phenotype, and combined prognostic models to assess whether phenotype membership improved prognostic performance beyond TSS.

## 2. Materials and Methods

### 2.1. Study Design

**Figure 1:**
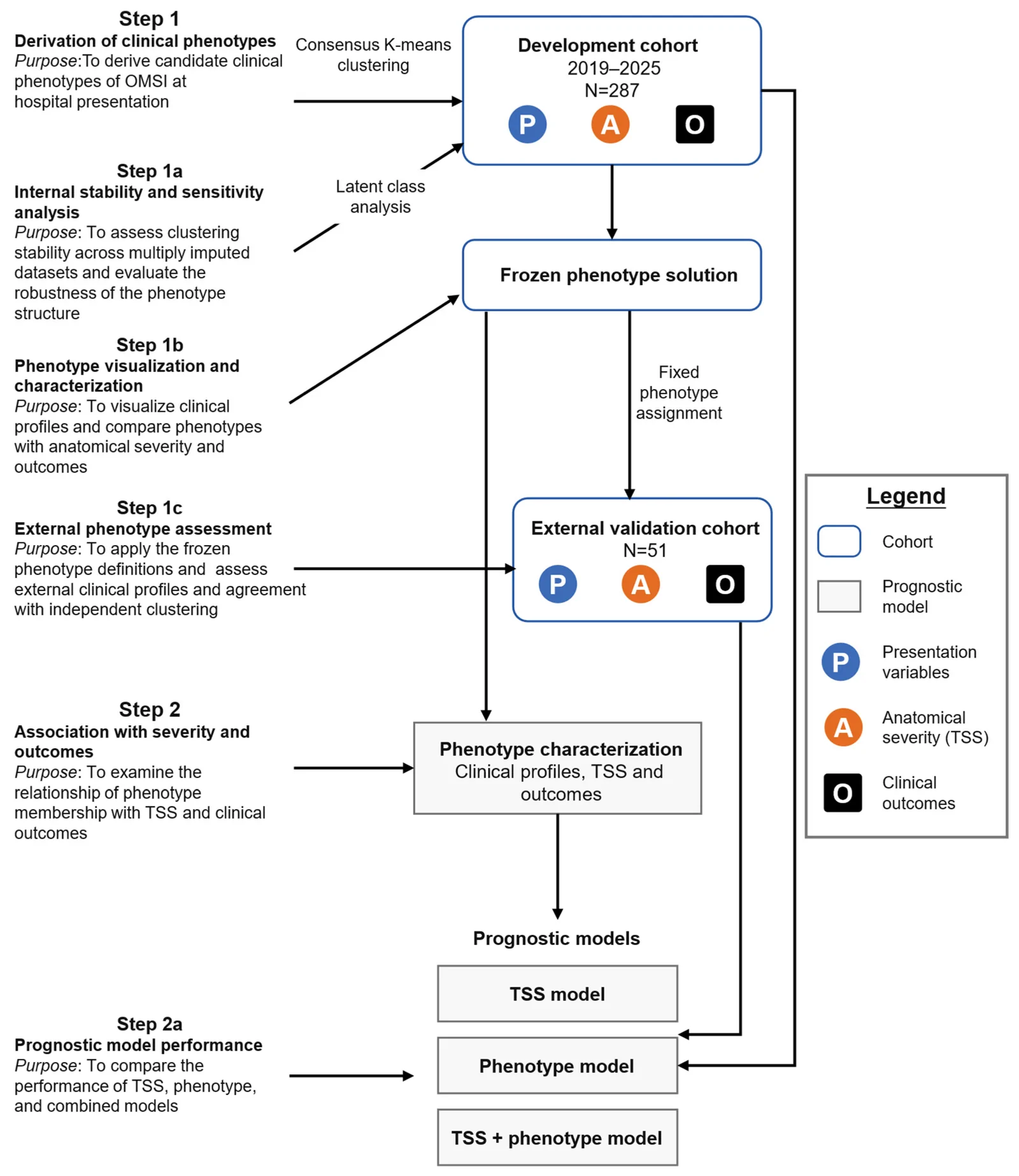
Study design and analytical workflow, showing derivation of candidate clinical phenotypes, internal stability assessment, external phenotype assessment, phenotype characterization, and prognostic evaluation. External assessment includes frozen phenotype assignment, description of clinical profiles, and comparison of frozen assignments with independent clustering results. Abbreviations: A, anatomical severity; O, clinical outcomes; OMSI, oral and maxillofacial space infection; P, presentation variables; TSS, traditional severity score.

This two-center retrospective cohort study included patients admitted with OMSI between January 2019 and June 2025. Patients treated at West China Hospital of Stomatology, Sichuan University, formed the development cohort. Patients treated at Panzhihua Central Hospital formed the external validation cohort. The overall study workflow is shown in Figure 1.

The study was conducted in accordance with the Declaration of Helsinki [14] and was approved by the Medical Ethics Committee of West China Hospital of Stomatology, Sichuan University (protocol code WCHSIRB-D-2026-152; approval date: 13 February 2026). This approval covered the retrospective analysis of clinical records collected during the study period. Clinical records were de-identified before analysis, and written informed consent was waived for this retrospective study.

### 2.2. Eligibility Criteria

Eligible patients met all of the following criteria: (1) a diagnosis of OMSI; (2) pretreatment computed tomography (CT) performed during the index hospitalization; and (3) clinical records and imaging data sufficient to confirm eligibility and ascertain the required study variables and outcomes.

Patients were excluded if they had (1) a malignant tumor or a history of radiotherapy or chemotherapy that could affect clinical assessment; (2) insufficient clinical records or pretreatment imaging to confirm eligibility or ascertain the required study variables; or (3) duplicate admissions during the study period. For patients with more than one eligible admission, only the first was retained. Patient selection is summarized in Figure S1.

### 2.3. Clinical Variables and Outcomes

We selected 24 variables based on their clinical relevance to OMSI and their routine availability in the electronic health record at hospital presentation. These included demographic variables (age, sex, and body mass index [BMI]); vital signs (temperature, heart rate, respiratory rate, and mean arterial pressure [MAP]); hematological variables (white blood cell count, platelet count, hemoglobin, and absolute monocyte count); inflammatory and coagulation markers (neutrophil-to-lymphocyte ratio [NLR], high-sensitivity C-reactive protein [hs-CRP], and fibrinogen); serum biochemical variables (potassium, sodium, chloride, calcium, total bilirubin, albumin, and glucose); and clinical features (trismus, dysphagia, and dyspnea). BMI, MAP, and NLR were calculated from their component variables. Detailed variable definitions and coding are provided in Supplementary Methods S1.

Anatomical involvement was assessed using pretreatment CT, and TSS was calculated from the involved fascial spaces according to the scoring system described by Flynn et al. [3,4]. Anatomical variables, TSS, treatment variables, and clinical outcomes were not included among the 24 clustering variables. However, anatomical space indicators were used as auxiliary predictors during multiple imputation, as detailed in Supplementary Methods S2. Anatomical severity and recorded clinical outcomes were then examined in relation to the development-derived phenotypes.

The primary composite outcome was defined as any of the following recorded during the index hospitalization: intensive care unit (ICU) admission, tracheostomy, septic shock, or descending mediastinal infection. ICU admission was determined by treating clinicians during routine care and identified from hospitalization records at both centers. The secondary composite outcome included a hospital stay longer than 14 days, repeat debridement, or use of special-grade antibiotics. The secondary outcome was summarized descriptively, and outcome models were fitted for the primary composite outcome.

### 2.4. Data Preprocessing

Variable distributions, missingness, and pairwise correlations were examined before phenotype derivation. Missing continuous baseline values were imputed using multiple imputation by chained equations with predictive mean matching, generating 20 completed datasets [15]. BMI, MAP, and NLR were recalculated from their component variables after imputation.

Right-skewed continuous variables were log-transformed before clustering. Continuous variables were standardized within each completed derivation dataset, whereas categorical variables retained their prespecified coding. For external validation, the same variable transformations and coding rules were used, and continuous variables were standardized using the corresponding parameters from the derivation cohort. Further details of imputation and preprocessing are provided in Supplementary Methods S2.

### 2.5. Phenotype Derivation and Internal Assessment

Ordering Points To Identify the Clustering Structure (OPTICS) reachability plots were first examined to characterize the underlying density structure [16]. Consensus k-means clustering was then performed separately in each of the 20 completed development datasets for K = 2–6 [17,18].

Candidate solutions were evaluated using the consensus matrix, the proportion of ambiguous clustering (PAC), the cumulative distribution function of consensus values, cluster consensus, silhouette width, phenotype size, and assignment confidence [16,17,18,19]. Agreement across imputed datasets was assessed using pairwise adjusted Rand indices (ARIs) and case-level assignment stability [20]. A mean within-cluster consensus of 0.80 was prespecified as the threshold for stable membership.

The number of phenotypes was selected based on clustering stability, agreement across imputations, assignment confidence, and phenotype size. Anatomical severity, clinical outcomes, treatment information, and data from the external validation cohort were not used to select the number of phenotypes. The selected phenotype solution was then frozen for subsequent characterization, external assignment, and prognostic analyses. Detailed definitions of the clustering metrics are provided in Supplementary Methods S3.

### 2.6. Phenotype Characterization and Sensitivity Analysis

Phenotype characteristics were summarized on their original clinical scales. Recorded diabetes history was also summarized by phenotype but was not included among the 24 clustering variables. Standardized profiles were used to visualize the multivariable patterns of each phenotype. Variables were also ranked by the mean pairwise absolute standardized difference between phenotypes. Principal component analysis was used to visualize patient-level phenotype patterns in two dimensions. Alluvial plots showed the distribution of TSS categories across phenotypes. TSS was also summarized within each phenotype using the median and interquartile range (IQR).

Latent class analysis (LCA) was performed as a sensitivity analysis using the same 24 presentation variables [21]. Continuous variables were categorized using tertile cutoffs derived from the development cohort, and the absent and mild trismus categories were combined before model fitting. Candidate LCA solutions were evaluated using the Bayesian information criterion, entropy, class size, clinical interpretability, and consistency across the 20 completed datasets. Additional details are provided in Supplementary Methods S4.

### 2.7. External Phenotype Assignment and Structural Assessment

The phenotype definitions and preprocessing procedures derived from the development cohort were fixed before external evaluation. Twenty completed external datasets were generated using the preprocessing rules and parameters derived from the development cohort. Within each completed dataset, each patient was assigned to the nearest development-derived phenotype centroid using Euclidean distance. Final phenotype membership was determined by majority vote across the 20 assignments.

The primary external analysis summarized phenotype sizes, agreement in frozen assignments across imputations, and phenotype-specific clinical profiles. Independent consensus k-means clustering was also performed in the external cohort for K = 2–6 as an exploratory structural assessment. At the K value selected in the development cohort, we used ARI to compare the independent external partition with the frozen phenotype assignments. Table S4 reports the independent clustering diagnostics. Supplementary Methods S5 provides detailed procedures and definitions of assignment confidence.

### 2.8. Prognostic Evaluation

Three prognostic models were fitted for the primary composite outcome using Firth’s mean bias-reduced logistic regression to reduce small-sample bias and address possible separation [22]. The models included TSS alone, phenotype membership alone, and their combination. TSS was modeled as a continuous variable in the TSS-only and combined models. Phenotype membership was entered as a categorical variable, with Phenotype 1 as the reference category. The TSS, phenotype, and combined models included 1, 3, and 4 predictor coefficients, respectively, excluding the intercept. Including the intercept, the phenotype and combined models had 4 and 5 coefficients, respectively.

Model discrimination was assessed using the area under the receiver operating characteristic curve (AUC). Overall predictive accuracy was assessed using the Brier score, and calibration was assessed using the calibration intercept and slope [23,24]. Net benefit was assessed using decision-curve analysis [25]. Internal validation used 1000 bootstrap resamples. Outcome models were refitted in each resample, and mean optimism was subtracted from their apparent performance. Phenotype assignments were held fixed throughout; multiple imputation, clustering, and phenotype selection were not repeated. We evaluated the frozen models in the external cohort using the same measures and the observed-to-expected (O/E) ratio. In an exploratory post hoc analysis, we recalibrated each model using its original predicted log-odds, updating the intercept alone or both the intercept and slope. The updated models were evaluated in the same 51 patients used for recalibration, and their apparent performance was reported separately from the original external validation. Supplementary Methods S6 provides further details.

### 2.9. Statistical Analysis

Continuous variables are presented as mean (standard deviation [SD]) or median (interquartile range [IQR]), according to their distributions. Categorical variables are presented as counts and percentages. Associations with the primary composite outcome were reported as odds ratios (ORs) with 95% confidence intervals (CIs). All tests were two-sided, with *p* < 0.05 indicating nominal statistical significance. Between-group comparisons were exploratory, and their *p* values were not adjusted for multiple testing. All analyses were performed using R version 4.5.2 (R Foundation for Statistical Computing, Vienna, Austria).

Supplementary Methods S1–S9 provide details on clinical variables and outcomes (S1), preprocessing (S2), phenotype derivation and selection (S3), phenotype characterization and sensitivity analyses (S4), external phenotype assignment (S5), prognostic and statistical analyses (S6), post hoc descriptive analyses of diabetes history, TSS, and between-center differences (S7), paired incremental prognostic performance analysis (S8), and glucose-exclusion sensitivity analysis (S9).

## 3. Results

### 3.1. Study Population

The development cohort included 287 patients, and the external validation cohort included 51 patients (Table 1; Figure S1). The primary composite outcome occurred in 35 of 287 patients (12.2%) in the development cohort and 22 of 51 patients (43.1%) in the external validation cohort. ICU admission occurred in 13 of 287 patients (4.5%) in the development cohort and 19 of 51 (37.3%) in the external cohort. Tracheostomy occurred in 20 of 287 (7.0%) and 4 of 51 (7.8%) patients, respectively. The secondary composite outcome occurred in 60 of 287 patients (20.9%) and 21 of 51 patients (41.2%), respectively. Baseline clinical characteristics, fascial-space involvement, and individual outcome components are shown in Table 1. A post hoc comparison also identified differences in selected clinical characteristics and data completeness between centers (Table S11). Before imputation, values for both glucose and hs-CRP were available for 257 of 287 patients (89.5%) in the development cohort and 23 of 51 patients (45.1%) in the external cohort.

### 3.2. Phenotype Derivation and Stability

**Figure 2:**
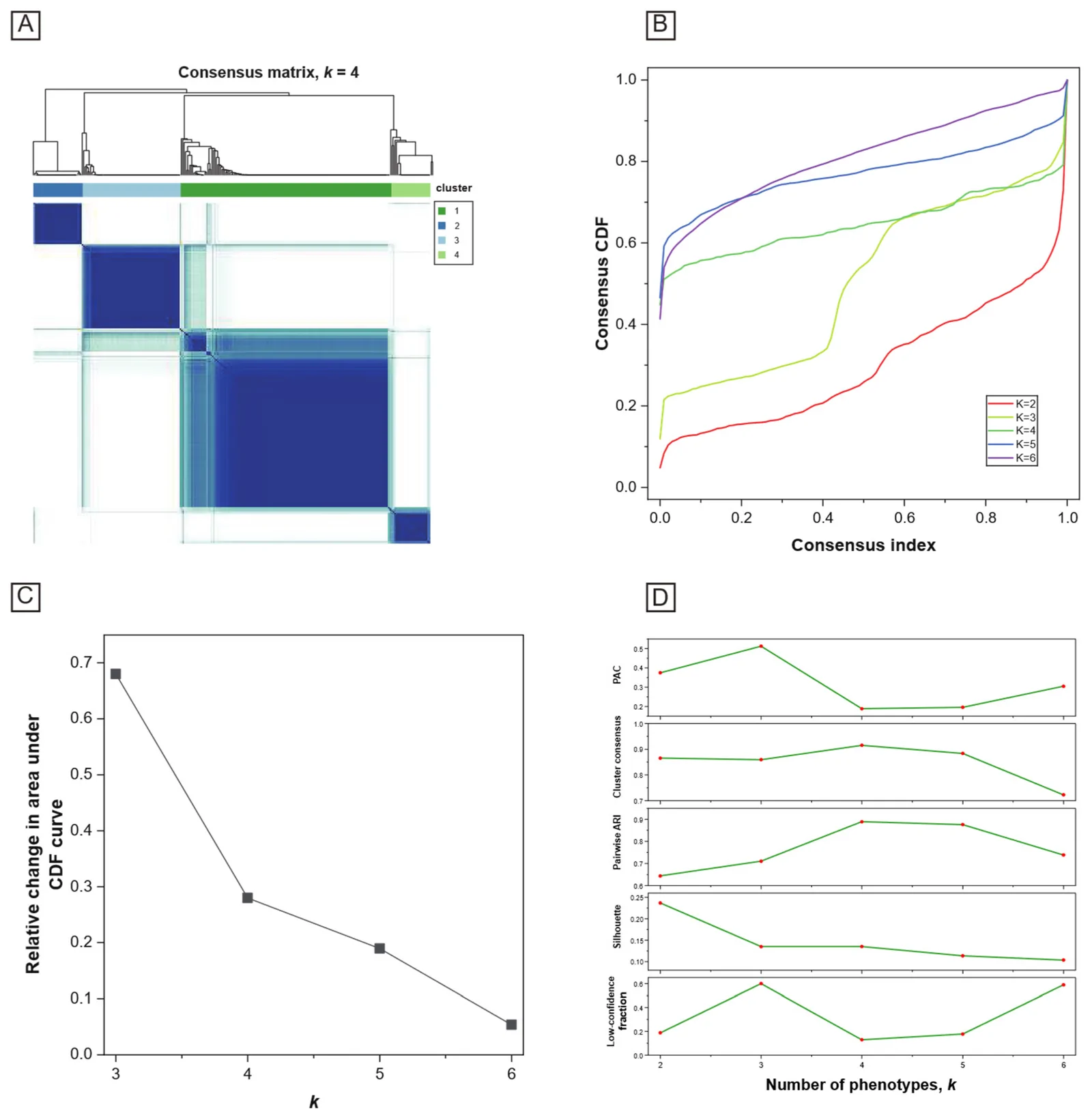
Consensus clustering diagnostics for phenotype derivation in the development cohort. (**A**) Consensus matrix for the selected phenotype solution; (**B**) consensus cumulative distribution function (CDF) curves across candidate cluster solutions; (**C**) relative change in CDF area between consecutive cluster solutions; (**D**) clustering diagnostics across candidate solutions, including the proportion of ambiguous clustering (PAC), cluster consensus, pairwise adjusted Rand index (ARI), silhouette width, and low-confidence fraction.

Among the candidate solutions from K = 2 to K = 6, the four-phenotype consensus k-means solution was retained for the main analysis (Figure 2; Tables S1 and S2). At K = 4, PAC was 0.188, mean cluster consensus was 0.915, mean pairwise ARI was 0.889, mean silhouette width was 0.135, and the low-confidence fraction was 12.9%. Phenotype 1 included 150 patients (52.3%), Phenotype 2 included 35 (12.2%), Phenotype 3 included 71 (24.7%), and Phenotype 4 included 31 (10.8%). At K = 5, PAC was 0.196, mean pairwise ARI was 0.876, and the low-confidence fraction was 17.8%. Group sizes were more balanced at K = 5 than at K = 4 (largest-to-smallest group ratio: 2.73 versus 4.84). In the LCA sensitivity analysis, the four-class solution had the lowest mean Bayesian information criterion (BIC, 13,783.324) and an entropy of 0.936 (Table S3; Figure S6). Compared with K = 5, K = 4 had higher mean cluster consensus (0.915 vs. 0.884) and a larger CDF-area increment (0.142 vs. 0.123; Table S1). Together with the other clustering diagnostics, these findings supported retaining K = 4 for the main analysis.

### 3.3. Clinical Characteristics of the Phenotypes

**Figure 3:**
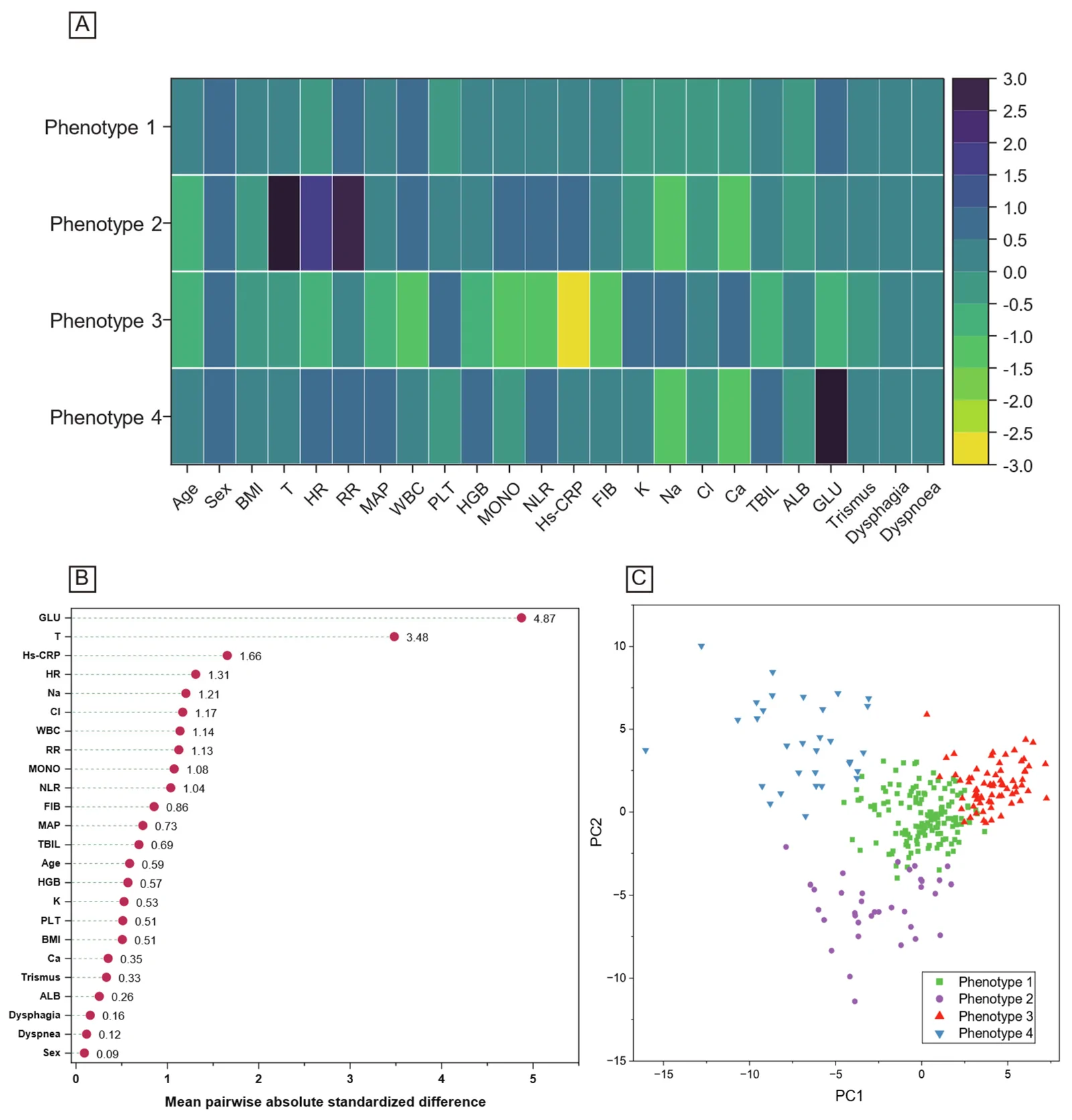
Clinical profiles of the four OMSI phenotypes in the development cohort. (**A**) Mean preprocessed feature values for each phenotype across 20 imputed datasets. For continuous variables, positive and negative values indicate values above and below the corresponding centering value, respectively. Trismus retains its prespecified ordinal coding, and binary variables are shown as proportions. The color scale ranges from −3 to +3. (**B**) Variables ranked by the mean pairwise absolute standardized difference between phenotypes; larger values indicate greater differences. (**C**) Principal component analysis (PCA) plot showing individual patients by phenotype. Each point represents one patient. PC1, principal component 1; PC2, principal component 2.

The four phenotypes showed different clinical profiles across the 24 presentation variables (Table 2 and Figure 3). Glucose showed the largest mean pairwise absolute standardized difference, followed by temperature, hs-CRP, heart rate, sodium, and chloride. Phenotype 1 showed intermediate values for most inflammatory variables. Phenotype 2 showed fever and elevated inflammatory markers, with a median temperature of 38.6 °C, heart rate of 112 beats/min, and hs-CRP level of 159.9 mg/L. Phenotype 3 had lower inflammatory marker levels, with median values of 7.73 × 10^9^/L for WBC, 3.59 for NLR, and 19.1 mg/L for hs-CRP. Phenotype 4 had higher glucose levels (median, 14.7 mmol/L) and lower sodium (136.0 mmol/L) and chloride (98.5 mmol/L) concentrations. The median hs-CRP level was 167.1 mg/L, and the median temperature was 36.5 °C. A history of diabetes was recorded in 29 of 31 patients (93.5%) in Phenotype 4 and 35 of 256 patients (13.7%) in Phenotypes 1–3 (Table 2). Among the 64 patients with a recorded history of diabetes in the development cohort, 29 (45.3%) were assigned to Phenotype 4 (Table S10). The PCA plot showed partial separation among the four phenotypes (Figure 3C). In a post hoc sensitivity analysis excluding glucose from clustering, the resulting K = 4 partition showed limited agreement with the original partition (ARI = 0.430), with particularly low agreement for the original Phenotype 4 (best-matched Jaccard coefficient = 0.158; Table S13).

### 3.4. Anatomical Severity and Outcomes

**Figure 4:**
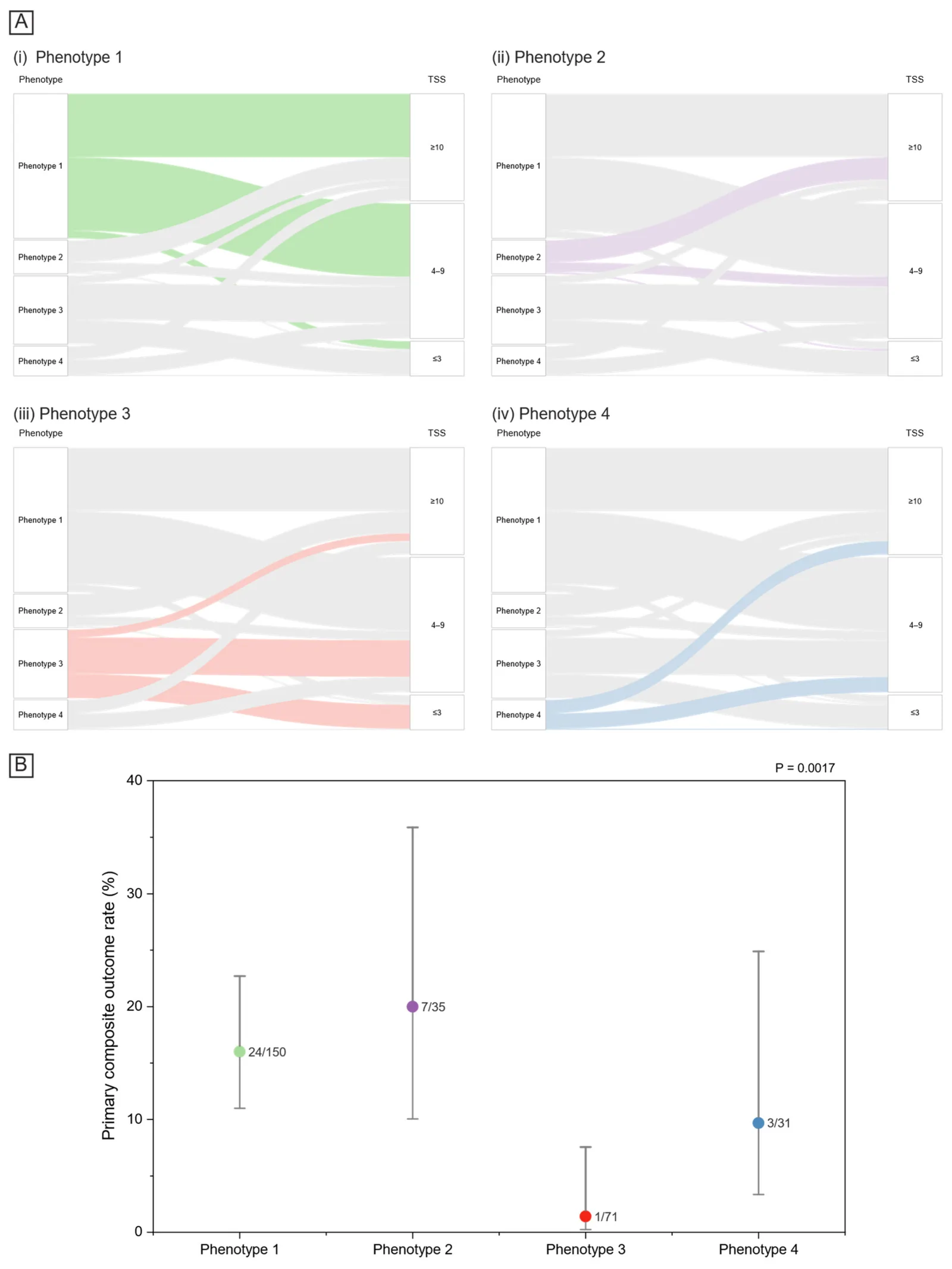
OMSI phenotypes in relation to anatomical severity and the primary composite outcome. (**A**) Alluvial plots showing the distribution of phenotypes across traditional severity score (TSS) categories (≤3, 4–9, and ≥10). TSS was calculated using Flynn’s scoring system [3,4]. (**B**) Primary composite outcome rates by phenotype in the development cohort (*p* = 0.0017). Error bars indicate 95% confidence intervals.

Each phenotype included patients from multiple TSS categories (Figure 4A). Median TSS values (IQRs) were 9 (7–12.75), 11 (7.5–14), 5 (3–8), and 9 (7–13) for Phenotypes 1–4, respectively. The primary composite outcome occurred in 24 of 150 patients (16.0%) in Phenotype 1, 7 of 35 (20.0%) in Phenotype 2, 1 of 71 (1.4%) in Phenotype 3, and 3 of 31 (9.7%) in Phenotype 4 (*p* = 0.0017; Figure 4B; Table S7).

In a post hoc descriptive analysis of patients with TSS values of 4–9 (*n* = 140), Phenotypes 1–4 included 76, 10, 38, and 16 patients, respectively. All four phenotypes had a median TSS of 7, but their temperature, glucose, hs-CRP, and NLR distributions differed (Table S10). As these variables were used for clustering, the comparisons were descriptive.

In the phenotype-only model, Phenotype 3 had lower odds of the primary composite outcome than Phenotype 1 (OR, 0.11; 95% CI, 0.02–0.59; *p* = 0.010). In the combined model, Phenotype 3 had an OR of 0.28 compared with Phenotype 1 (95% CI, 0.05–1.59; *p* = 0.150). Each 1-point increase in TSS was associated with higher odds of the primary composite outcome (OR, 1.26; 95% CI, 1.15–1.38; *p* < 0.001) (Table S6). The association for Phenotype 3 was attenuated after adjustment for continuous TSS.

### 3.5. External Phenotype Assignment and Assessment

The frozen procedure assigned 25, 6, 16, and 4 patients in the external cohort to Phenotypes 1–4, respectively. Two patients (3.9%) had low-confidence assignments, defined by a maximum vote proportion below 0.80 across the 20 imputed datasets.

Independent clustering at K = 4 showed limited agreement with the frozen assignments (ARI = 0.320). Within this independent analysis, the mean pairwise ARI across the 20 imputed datasets was 0.754, and the mean within-cluster consensus was 0.815. The low-confidence fraction was 29.4%, with low confidence defined as within-cluster consensus below 0.80 or a consensus margin below 0.10 (Table S4).

Table S5 summarizes external phenotype characteristics, and Figures S4 and S5 compare clinical profiles across cohorts. After frozen assignment in the external cohort, Phenotype 2 had the highest temperature and hs-CRP levels. Phenotype 3 had lower white blood cell counts, NLR values, and hs-CRP levels. Phenotype 4 had the highest glucose levels and lower sodium and chloride concentrations. All four patients in Phenotype 4 had a recorded history of diabetes, compared with 11 of 47 patients (23.4%) in Phenotypes 1–3 (Table S5). Among the 15 external patients with a recorded history of diabetes, four (26.7%) were assigned to Phenotype 4 (Table S10).

### 3.6. Prognostic Model Performance

#### 3.6.1. Development Cohort

**Figure 5:**
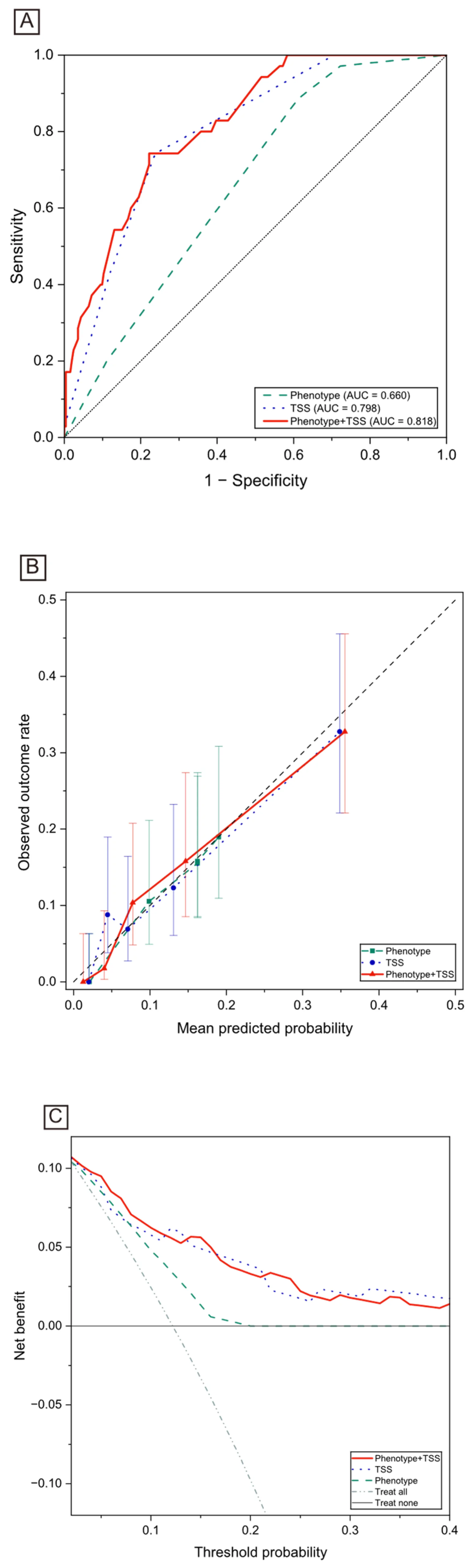
Apparent prognostic performance of the TSS, phenotype, and combined models in the development cohort. (**A**) Receiver operating characteristic (ROC) curves for the three models. (**B**) Calibration plots comparing predicted and observed outcome probabilities. The diagonal line indicates perfect calibration. (**C**) Decision-curve analysis showing net benefit across threshold probabilities, with treat-all and treat-none strategies as references.

In the development cohort, the TSS, phenotype, and combined models had apparent AUCs of 0.798 (95% CI, 0.726–0.878), 0.660 (95% CI, 0.589–0.730), and 0.818 (95% CI, 0.753–0.884), respectively. The corresponding Brier scores were 0.088, 0.103, and 0.088 (Table 3; Figure 5). After bootstrap optimism correction, the AUCs were 0.798, 0.631, and 0.799, respectively. The apparent gain in discrimination from adding phenotype membership to TSS was largely lost after correction. The paired difference in optimism-corrected AUC between the combined and TSS-only models was 0.0015 (95% CI, −0.0144 to 0.0498; Table S12).

#### 3.6.2. External Cohort

The original external validation used the frozen models. AUCs were 0.926 (95% CI, 0.850–0.981) for the TSS model, 0.665 (95% CI, 0.513–0.796) for the phenotype model, and 0.891 (95% CI, 0.785–0.971) for the combined model. Brier scores were 0.301, 0.328, and 0.303, respectively. The paired AUC difference for the combined model versus the TSS-only model was −0.0345 (95% CI, −0.0886 to 0.0042; Table S12).

For the TSS, phenotype, and combined models, calibration intercepts were 2.537, 1.926, and 2.531, and calibration slopes were 2.282, 0.549, and 1.464, respectively. O/E ratios were 4.425, 3.620, and 4.424, indicating substantial underestimation of absolute risk by all three models. Table S8 reports net benefit at threshold probabilities of 10%, 20%, 30%, and 40%.

We performed exploratory recalibration and assessed the updated models’ apparent performance in the same 51 patients. Intercept-only recalibration reduced Brier scores to 0.129, 0.235, and 0.144 for the TSS, phenotype, and combined models, respectively. Updating both the intercept and slope yielded Brier scores of 0.114, 0.228, and 0.138, respectively. Both approaches yielded O/E ratios close to 1, with unchanged AUCs (Table S9).

## 4. Discussion

In this two-center retrospective study, we derived four candidate clinical phenotypes of OMSI from routinely available data at hospital presentation and evaluated their external reproducibility and prognostic value beyond TSS. The phenotypes showed different inflammatory, physiological, and metabolic profiles, although their clinical characteristics overlapped. Independent external clustering showed limited agreement with the frozen assignments, and adding phenotype membership to TSS did not consistently improve prognostic discrimination. These findings distinguish the descriptive value of the candidate phenotypes from the evidence needed to establish a reproducible and clinically useful classification.

In the development cohort, Phenotypes 2 and 4 both showed elevated inflammatory markers, with median hs-CRP levels of 159.9 and 167.1 mg/L and median NLR values of 12.50 and 11.92, respectively. However, their temperature and glucose profiles differed substantially: median temperature was 38.6 °C in Phenotype 2 and 36.5 °C in Phenotype 4, whereas median glucose was 6.0 and 14.7 mmol/L, respectively. These differences characterize the combinations of clinical measurements observed within the derived groups. Previous studies have established associations of inflammatory markers, diabetes, anatomical disease extent, and airway-related symptoms with clinically important outcomes in odontogenic, maxillofacial, and cervicofacial infections [7,8,9,12,13]. Our analysis extends this descriptive perspective by examining how routinely recorded characteristics occur together in individual patients and by evaluating whether the resulting groups can be reproduced and provide additional prognostic information.

Phenotype 4 was characterized by high glucose and relatively lower sodium and chloride concentrations. A history of diabetes was recorded in 29 of 31 patients (93.5%) in this phenotype in the development cohort and all four patients in the external cohort. However, these 29 patients accounted for 45.3% of the 64 development patients with a recorded history of diabetes. Excluding glucose from clustering substantially changed the membership of Phenotype 4 (Table S13), highlighting its dependence on the glucose variable. Together, these findings describe a glucose-dominant clinical profile enriched for diabetes history. A prospective study of cervicofacial infections reported different associations of diabetes and stress hyperglycemia with disease severity and clinical outcomes [26]. Without HbA1c measurements and information on glucose-lowering treatment, the relative contributions of pre-existing dysglycemia and acute stress could not be determined [27]. Fluid-status data were also unavailable, limiting interpretation of the lower sodium and chloride concentrations. Previous studies have reported associations between electrolyte abnormalities and outcomes in severe infection [28,29].

Anatomical spread remains central to severity assessment in OMSI [3,4,6]. The derived phenotypes included patients across multiple TSS categories, although anatomical variables and TSS were not directly used as clustering features. In the development cohort, Phenotypes 1 and 4 had the same median TSS (9) but different inflammatory and metabolic profiles. Among patients with TSS values of 4–9, all four phenotypes had a median TSS of 7, while the distributions of the clinical variables used for clustering differed (Table S10). These observations describe variation in systemic presentation within broad categories of anatomical severity. Phenotype 3 had the lowest overall median TSS (5), and its association with the primary composite outcome was attenuated after adjustment for continuous TSS (adjusted OR, 0.28; 95% CI, 0.05–1.59). This attenuation suggests that anatomical severity may contribute to the observed phenotype–outcome association.

Adding phenotype membership to TSS did not consistently improve prognostic discrimination. In the development cohort, the combined model had a slightly higher apparent AUC than TSS alone (0.818 vs. 0.798), but the difference was small after optimism correction (paired ΔAUC = 0.0015; 95% CI, −0.0144 to 0.0498). In the external cohort, the combined model had a lower AUC than TSS alone (0.891 vs. 0.926; paired ΔAUC = −0.0345; 95% CI, −0.0886 to 0.0042). The phenotype-only model showed limited discrimination, with an optimism-corrected AUC of 0.631 in the development cohort and an external AUC of 0.665. Both paired confidence intervals included zero (Table S12).

The four-phenotype solution was retained based on clustering stability and assignment quality in the development cohort. However, the low mean silhouette width (0.135) indicated overlapping patient profiles, and several clustering metrics were similar for K = 4 and K = 5. The four-class LCA model had the lowest mean BIC, but this analysis used the same patients and underlying clinical variables and did not provide independent confirmation. In the external cohort, independent K = 4 clustering showed limited agreement with the frozen assignments (ARI = 0.320). Clinical patterns observed after frozen assignment described external patients within the development-derived classification. Similar challenges in reproducing clinical phenotypes across hospitals have been reported in sepsis research [30]. Together, these findings indicate uncertainty about the cross-center reproducibility of the four-phenotype structure.

The recorded primary composite outcome occurred in 12.2% of patients in the development cohort and 43.1% in the external cohort. ICU admission was substantially more frequent in the external cohort (37.3% vs. 4.5%), whereas tracheostomy rates were similar (7.8% vs. 7.0%). In the post hoc center comparison, the external cohort had lower median TSS and heart rate, higher respiratory rate, and a higher proportion of patients with recorded diabetes (Table S11). The completeness of admission laboratory measurements also differed between cohorts. Differences in case mix, referral patterns, data completeness, and ICU admission practices may have contributed to the variation in outcome rates and model calibration, although their relative contributions could not be determined. All three frozen models substantially underestimated absolute risk in the external cohort, with O/E ratios of 4.425, 3.620, and 4.424 for the TSS, phenotype, and combined models, respectively.

Differences in case mix and clinical practice can affect model calibration across hospitals [23,24,31,32]. The phenotype model’s external calibration slope was imprecisely estimated (0.549; 95% CI, −0.008 to 1.417). Both recalibration approaches reduced Brier scores in the same 51 patients used for updating (Table S9). With maximum-likelihood logistic recalibration and a freely estimated intercept, the mean predicted risk matched the observed event rate, yielding O/E ratios close to 1. Positive updating slopes preserved the ordering of predicted risks, so AUCs remained unchanged. These estimates represent the apparent performance of the recalibrated models.

Previous studies of OMSI and cervicofacial infections have linked anatomical involvement, airway-related symptoms, inflammatory markers, and metabolic comorbidity with important clinical outcomes [12,13]. Our findings describe how routinely recorded inflammatory, physiological, and metabolic features occur together, including among patients within the same broad TSS category. These candidate phenotypes provide a way to characterize differences in clinical presentation and case mix alongside anatomical severity. However, limited agreement with independent external clustering and the absence of consistent prognostic improvement beyond TSS do not support using the current classification for clinical triage, treatment selection, or individualized risk prediction. Future studies using standardized measurements should assess whether more reproducible clinical patterns can provide useful information for patient assessment and management.

### Limitations

The retrospective design limited the analysis to variables routinely recorded at hospital presentation and may have introduced selection and information bias. The timing of individual outcome components relative to baseline assessment could not be consistently established. The prognostic analyses therefore describe associations with the recorded primary composite outcome, which may include severe conditions already present at admission and complications developing later during hospitalization. The development cohort included 35 primary outcome events, with one among 71 patients in Phenotype 3 and three among 31 patients in Phenotype 4 (Table S7). Firth’s regression was used to reduce small-sample estimation bias, but the small number of events limited the precision of phenotype-specific estimates. During bootstrap validation, the outcome models were refitted with the final phenotype assignments held fixed. This procedure did not account for uncertainty from multiple imputation, clustering, or selection of the number of phenotypes. The additional paired performance intervals were also estimated with phenotype assignments held fixed.

Diabetes history was recorded, but the analysis dataset lacked HbA1c measurements, detailed comorbidity information, and data on glucose-lowering treatment and fluid status. These data gaps limited assessment of the relative contributions of pre-existing dysglycemia and acute physiological changes to the metabolic profile of Phenotype 4. Changes in phenotype profiles over time could not be assessed because longitudinal clinical measurements were unavailable. Microbiological findings, immune biomarkers, and molecular data were also unavailable, limiting investigation of the mechanisms underlying the observed clinical patterns. The glucose-exclusion sensitivity analysis removed glucose from the clustering variables but retained the original imputed datasets, which may still reflect glucose-related associations from the imputation process.

Both cohorts were drawn from hospitals in China, and the phenotypes were derived at a specialized tertiary oral healthcare center. Referral systems, case mix, and treatment practices may differ in other settings. The external cohort included 51 patients, limiting the precision of calibration estimates and our ability to assess structural reproducibility. Within this cohort, Phenotypes 2 and 4 included six and four patients, respectively, further limiting the precision of phenotype-specific estimates. Observed values for both glucose and hs-CRP were available in 23 of 51 external patients. The dataset lacked individual measurement timestamps and sufficient assay details to assess measurement comparability between centers, further limiting the interpretation of external phenotype and calibration results.

## 5. Conclusions

In this exploratory two-center retrospective study, we identified four candidate clinical phenotypes of OMSI with different inflammatory, physiological, and metabolic profiles. The groups showed overlapping clinical characteristics and differed in recorded diabetes history and anatomical severity. Independent external clustering showed limited agreement with the frozen assignments, and adding phenotype membership to TSS did not consistently improve prognostic discrimination. All three frozen models also underestimated absolute risk in the external cohort. These findings characterize clinical heterogeneity at presentation but do not establish a classification suitable for guiding clinical decisions. Larger multicenter studies using standardized measurements and outcome definitions are needed to assess phenotype reproducibility and clinical value.

## Figures and Tables

**Table 1:** Clinical characteristics, fascial-space involvement, and outcomes in the development and external validation cohorts.

Variable		Development Cohort	External Validation Cohort	*p*
No.		287	51	
Demographic				
	Age, years, median (IQR)	55.0 (41.0–68.0)	60.0 (50.5–73.0)	0.076
	Sex, *n* (%)			0.428
	Male	180 (62.7%)	29 (56.9%)	
	Female	107 (37.3%)	22 (43.1%)	
	Height, cm, median (IQR)	160.0 (155.0–168.0)	161.6 (153.8–169.6)	0.996
	Weight, kg, median (IQR)	60.0 (51.0–67.0)	59.9 (53.4–67.7)	0.618
Vital signs				
	Temperature, °C, median (IQR)	36.6 (36.4–37.0)	36.4 (36.2–36.6)	0.005
	Heart rate, beats/min, median (IQR)	93.0 (81.0–105.0)	84.0 (75.0–96.0)	0.003
	Respiratory rate, breaths/min, median (IQR)	18.0 (18.0–20.0)	20.0 (19.0–20.0)	<0.001
	Systolic blood pressure, mmHg, median (IQR)	121.0 (110.0–136.0)	124.0 (112.5–138.0)	0.827
	Diastolic blood pressure, mmHg, median (IQR)	77.0 (70.0–86.0)	79.0 (69.0–85.0)	0.858
Clinical symptoms				
	Trismus, *n* (%)			<0.001
	Absent	12 (4.2%)	10 (19.6%)	
	Mild	4 (1.4%)	9 (17.6%)	
	Moderate	68 (23.7%)	14 (27.5%)	
	Severe	203 (70.7%)	18 (35.3%)	
	Dysphagia, *n* (%)	84 (29.3%)	6 (11.8%)	0.009
	Dyspnea, *n* (%)	40 (13.9%)	9 (17.6%)	0.488
Laboratory				
	WBC, ×10^9^/L, median (IQR)	12.67 (9.08–16.36)	12.80 (10.07–15.64)	0.985
	PLT, ×10^9^/L, median (IQR)	249.0 (191.5–315.0)	236.0 (181.5–301.5)	0.247
	RBC, ×10^12^/L, median (IQR)	4.40 (3.96–4.84)	4.57 (4.14–5.01)	0.133
	HGB, g/L, mean (SD)	130.5 (20.1)	137.3 (22.0)	0.046
	NEUT, ×10^9^/L, median (IQR)	9.98 (6.93–13.89)	10.28 (7.43–12.46)	0.647
	LYMPH, ×10^9^/L, median (IQR)	1.27 (0.83–1.67)	1.42 (0.90–2.07)	0.143
	MONO, ×10^9^/L, median (IQR)	0.89 (0.61–1.16)	0.89 (0.60–1.19)	0.998
	K, mmol/L, median (IQR)	3.80 (3.55–4.12)	3.88 (3.56–4.10)	0.907
	Na, mmol/L, median (IQR)	138.34 (136.13–139.98)	138.70 (136.45–141.05)	0.282
	Cl, mmol/L, median (IQR)	101.50 (98.75–104.28)	102.00 (98.90–104.75)	0.731
	Ca, mmol/L, mean (SD)	2.22 (0.15)	2.18 (0.13)	0.058
	TBIL, μmol/L, median (IQR)	13.7 (10.3–18.8)	13.4 (9.3–23.4)	0.846
	ALB, g/L, mean (SD)	38.6 (5.1)	38.8 (5.8)	0.827
	hs-CRP, mg/L, median (IQR)	87.3 (40.9–158.8)	84.2 (37.0–143.8)	0.554
	GLU, mmol/L, median (IQR)	5.89 (5.12–7.66)	6.10 (5.09–7.33)	0.967
	FIB, g/L, median (IQR)	6.70 (5.25–7.49)	4.99 (4.46–6.13)	<0.001
Infection space				
	Submandibular space, *n* (%)	217 (75.6%)	42 (82.4%)	0.294
	Masseteric space, *n* (%)	191 (66.6%)	14 (27.5%)	<0.001
	Pterygomandibular space, *n* (%)	170 (59.2%)	11 (21.6%)	<0.001
	Parapharyngeal space, *n* (%)	156 (54.4%)	25 (49.0%)	0.481
	Buccal space, *n* (%)	132 (46.0%)	11 (21.6%)	0.001
	Infratemporal space, *n* (%)	74 (25.8%)	2 (3.9%)	<0.001
	Submental space, *n* (%)	60 (20.9%)	21 (41.2%)	0.002
	Temporal space, *n* (%)	64 (22.3%)	5 (9.8%)	0.041
	Infraorbital space, *n* (%)	42 (14.6%)	4 (7.8%)	0.192
	Sublingual space, *n* (%)	39 (13.6%)	9 (17.6%)	0.444
	Retropharyngeal space, *n* (%)	29 (10.1%)	10 (19.6%)	0.050
Clinical severity and outcomes				
	Primary composite outcome, *n* (%)	35 (12.2%)	22 (43.1%)	<0.001
	ICU admission, *n* (%)	13 (4.5%)	19 (37.3%)	<0.001
	Tracheostomy, *n* (%)	20 (7.0%)	4 (7.8%)	0.770
	Septic shock, *n* (%)	0 (0.0%)	2 (3.9%)	0.022
	Descending mediastinal infection, *n* (%)	3 (1.0%)	2 (3.9%)	0.165
	Secondary composite outcome, *n* (%)	60 (20.9%)	21 (41.2%)	0.002
	Hospitalization > 14 days, *n* (%)	44 (15.3%)	14 (27.5%)	0.043
	Repeated debridement, *n* (%)	17 (5.9%)	10 (19.6%)	0.003
	Special-grade antibiotic use, *n* (%)	14 (4.9%)	11 (21.6%)	<0.001

Abbreviations: ALB, albumin; Ca, calcium; Cl, chloride; FIB, fibrinogen; GLU, glucose; HGB, hemoglobin; hs-CRP, high-sensitivity C-reactive protein; ICU, intensive care unit; IQR, interquartile range; K, potassium; LYMPH, absolute lymphocyte count; MONO, absolute monocyte count; Na, sodium; NEUT, absolute neutrophil count; PLT, platelet count; RBC, red blood cell count; SD, standard deviation; TBIL, total bilirubin; WBC, white blood cell count.

**Table 2:** Presentation characteristics of the four OMSI phenotypes in the development cohort.

Characteristic		Total	Phenotype			
			1	2	3	4
	Participants, *n*	287	150	35	71	31
Demographic variables						
	Male sex, *n* (%)	180 (62.7%)	98 (65.3%)	23 (65.7%)	37 (52.1%)	22 (71.0%)
	Age, years, median (IQR)	55.0 (41.0, 68.0)	59.0 (48.0, 69.0)	52.0 (22.0, 66.0)	53.0 (30.0, 67.0)	53.0 (51.0, 68.5)
	BMI, kg/m^2^, median (IQR)	22.5 (20.1, 24.8)	23.1 (21.1, 25.1)	21.3 (18.2, 24.6)	21.5 (18.8, 23.8)	22.8 (21.0, 24.4)
Vital signs						
	Temperature, °C, median (IQR)	36.6 (36.4, 37.0)	36.6 (36.4, 36.9)	38.6 (38.1, 39.0)	36.5 (36.3, 36.7)	36.5 (36.3, 36.8)
	Heart rate, beats/min, median (IQR)	93 (81, 105)	91 (80, 101)	112 (104, 124)	89 (78, 96)	101 (90, 112)
	Respiratory rate, breaths/min, median (IQR)	18 (18, 20)	18 (18, 19)	20 (19, 22)	18 (18, 18)	19 (18, 20)
	Mean arterial pressure, mmHg, median (IQR)	92.3 (84.8, 101.5)	94.2 (87.3, 105.0)	94.3 (83.3, 106.2)	86.7 (78.3, 92.0)	97.0 (88.5, 106.2)
Hematological measures						
	WBC, ×10^9^/L, median (IQR)	12.67 (9.08, 16.36)	13.87 (11.53, 16.81)	16.52 (12.57, 18.81)	7.73 (6.38, 9.34)	13.67 (10.23, 17.80)
	PLT, ×10^9^/L, median (IQR)	249 (192, 315)	230 (185, 297)	245 (182, 299)	297 (220, 360)	228 (188, 302)
	HGB, g/L, mean (SD)	130.5 (20.1)	132.5 (20.3)	131.3 (15.1)	123.0 (20.3)	137.4 (19.3)
	MONO, ×10^9^/L, median (IQR)	0.89 (0.62, 1.16)	1.02 (0.80, 1.27)	1.03 (0.82, 1.54)	0.59 (0.43, 0.75)	0.94 (0.54, 1.18)
Inflammatory and coagulation markers						
	NLR, median (IQR)	7.81 (4.36, 14.02)	9.80 (6.37, 16.44)	12.50 (6.98, 20.65)	3.59 (2.62, 4.99)	11.92 (8.44, 18.96)
	hs-CRP, mg/L, median (IQR)	87.3 (40.9, 158.8)	117.5 (70.6, 159.8)	159.9 (79.2, 213.7)	19.1 (5.2, 36.3)	167.1 (69.3, 224.1)
	FIB, g/L, median (IQR)	6.70 (5.26, 7.49)	7.07 (5.78, 7.89)	7.07 (6.14, 7.89)	5.07 (3.87, 6.20)	6.37 (6.06, 7.89)
Serum biochemical measures						
	K, mmol/L, median (IQR)	3.80 (3.55, 4.12)	3.74 (3.48, 4.05)	3.75 (3.46, 4.05)	4.00 (3.71, 4.27)	3.82 (3.66, 4.22)
	Na, mmol/L, median (IQR)	138.3 (136.1, 140.0)	138.4 (136.3, 139.9)	136.2 (134.3, 137.9)	139.0 (138.2, 141.0)	136.0 (133.0, 138.5)
	Cl, mmol/L, median (IQR)	101.5 (98.8, 104.3)	101.1 (98.7, 103.7)	99.1 (96.3, 101.2)	104.2 (102.4, 105.7)	98.5 (96.0, 102.2)
	Ca, mmol/L, mean (SD)	2.22 (0.15)	2.21 (0.16)	2.21 (0.14)	2.27 (0.13)	2.20 (0.15)
	TBIL, µmol/L, median (IQR)	13.7 (10.3, 18.8)	14.9 (11.8, 19.4)	14.7 (11.3, 21.0)	10.3 (8.2, 13.8)	16.4 (11.0, 21.3)
	ALB, g/L, mean (SD)	38.6 (5.1)	38.3 (5.1)	38.6 (5.0)	39.6 (5.0)	37.9 (5.2)
	GLU, mmol/L, median (IQR)	5.9 (5.1, 7.7)	6.0 (5.3, 7.4)	6.0 (5.2, 7.2)	5.2 (4.8, 5.7)	14.7 (13.2, 17.4)
Presenting symptoms						
	Trismus, *n* (%)					
	Absent	12 (4.2%)	2 (1.3%)	2 (5.7%)	7 (9.9%)	1 (3.2%)
	Mild	4 (1.4%)	1 (0.7%)	1 (2.9%)	2 (2.8%)	0 (0.0%)
	Moderate	68 (23.7%)	36 (24.0%)	4 (11.4%)	24 (33.8%)	4 (12.9%)
	Severe	203 (70.7%)	111 (74.0%)	28 (80.0%)	38 (53.5%)	26 (83.9%)
	Dysphagia, *n* (%)	84 (29.3%)	51 (34.0%)	14 (40.0%)	7 (9.9%)	12 (38.7%)
	Dyspnea, *n* (%)	40 (13.9%)	21 (14.0%)	10 (28.6%)	4 (5.6%)	5 (16.1%)
Clinical history						
	Recorded diabetes history, *n* (%)	64 (22.3%)	21 (14.0%)	3 (8.6%)	11 (15.5%)	29 (93.5%)

Recorded diabetes history was not included among the 24 variables used for phenotype derivation. Abbreviations: ALB, albumin; BMI, body mass index; Ca, calcium; Cl, chloride; FIB, fibrinogen; GLU, glucose; HGB, hemoglobin; hs-CRP, high-sensitivity C-reactive protein; IQR, interquartile range; K, potassium; MONO, absolute monocyte count; Na, sodium; NLR, neutrophil-to-lymphocyte ratio; OMSI, oral and maxillofacial space infection; PLT, platelet count; SD, standard deviation; TBIL, total bilirubin; WBC, white blood cell count.

**Table 3:** Performance of the TSS, phenotype, and combined models in the development and external validation cohorts.

Validation Setting	Model	AUC (95% CI)	Brier Score	Calibration Intercept (95% CI)	Calibration Slope (95% CI)	O/E Ratio
Development cohort	TSS model	0.798 (0.726, 0.878)	0.088	−0.019 (−0.415, 0.342)	1.021 (0.711, 1.404)	0.986
	Phenotype model	0.660 (0.589, 0.730)	0.103	−0.050 (−0.424, 0.278)	1.149 (0.586, 2.796)	0.959
	Combined model	0.818 (0.753, 0.884)	0.088	−0.057 (−0.446, 0.307)	1.059 (0.774, 1.437)	0.959
External validation cohort	TSS model	0.926 (0.850, 0.981)	0.301	2.537 (1.962, 3.046)	2.282 (1.551, 4.668)	4.425
	Phenotype model	0.665 (0.513, 0.796)	0.328	1.926 (1.270, 2.565)	0.549 (−0.008, 1.417)	3.620
	Combined model	0.891 (0.785, 0.971)	0.303	2.531 (1.936, 3.143)	1.464 (0.861, 3.507)	4.424

Bootstrap optimism-corrected AUCs were calculated with the final phenotype assignments held fixed and are reported separately in Section 3.6.1. External cohort estimates were obtained from the original frozen models without recalibration. The observed-to-expected (O/E) ratio was calculated as the number of observed events divided by the sum of predicted probabilities in each cohort. Abbreviations: AUC, area under the receiver operating characteristic curve; CI, confidence interval; O/E, observed-to-expected ratio; TSS, traditional severity score.

## Data Availability

The original contributions presented in this study are included in the article and Supplementary Materials. Further inquiries can be directed to the corresponding authors.

## Supplementary Materials

The following supporting information can be downloaded at https://www.mdpi.com/article/10.3390/jcm15218414/s1. Supplementary Methods S1–S9; Table S1: Consensus clustering diagnostics for candidate solutions in the development cohort; Table S2: Assignment quality and phenotype sizes for candidate clustering solutions in the development cohort; Table S3: Latent class analysis diagnostics for candidate solutions in the development cohort; Table S4: Exploratory independent consensus clustering diagnostics in the external cohort (*n* = 51); Table S5: Presentation characteristics of the four OMSI phenotypes in the external validation cohort; Table S6: Associations of phenotype membership and TSS with the primary composite outcome in the development cohort; Table S7: Primary composite outcome by phenotype in the development and external validation cohorts; Table S8: External validation performance and decision-curve net benefit of the TSS, phenotype, and combined models; Table S9: Exploratory recalibration of frozen models in the external cohort; Table S10: Overlap of candidate phenotypes with recorded diabetes and anatomical severity; Table S11: Selected differences in clinical characteristics and data completeness between the two centers; Table S12: Paired differences in prognostic performance between the combined and TSS-only models; Table S13: Sensitivity of the original four-group partition to exclusion of glucose. Figure S1: Patient selection flow diagram; Figure S2: Correlation heatmap of baseline clinical variables assessed during preprocessing; Figure S3: OPTICS reachability plots across 20 multiply imputed datasets; Figure S4: Cross-cohort comparison of phenotype profiles: age through hemoglobin; Figure S5: Cross-cohort comparison of phenotype profiles: absolute monocyte count through glucose; Figure S6: Posterior class-membership probabilities for the four-class latent class model in the development cohort (*n* = 287).

## Abbreviations

The following abbreviations are used in this manuscript:

ALB	Albumin
ARI	Adjusted Rand index
AUC	Area under the receiver operating characteristic curve
BIC	Bayesian information criterion
BMI	Body mass index
CDF	Cumulative distribution function
CI	Confidence interval
CRP	C-reactive protein
CT	Computed tomography
FIB	Fibrinogen
GLU	Glucose
HbA1c	Hemoglobin A1c
HGB	Hemoglobin
HR	Heart rate
hs-CRP	High-sensitivity C-reactive protein
ICU	Intensive care unit
IQR	Interquartile range
LCA	Latent class analysis
LYMPH	Absolute lymphocyte count
MAP	Mean arterial pressure
MONO	Absolute monocyte count
NEUT	Absolute neutrophil count
NLR	Neutrophil-to-lymphocyte ratio
O/E	Observed-to-expected ratio
OMSI	Oral and maxillofacial space infection
OPTICS	Ordering Points To Identify the Clustering Structure
OR	Odds ratio
PAC	Proportion of ambiguous clustering
PCA	Principal component analysis
PLT	Platelet count
RBC	Red blood cell count
RR	Respiratory rate
SD	Standard deviation
TBIL	Total bilirubin
TSS	Traditional severity score
WBC	White blood cell count
