# Supplementary material for: Clinical Phenotypes of Oral and Maxillofacial Space Infection: Derivation, External Assessment, and Prognostic Evaluation
Source: J Clin Med. 2026 Oct 29;15(21):8414. doi: 10.3390/jcm15218414 (PMC13763910; doi:10.3390/jcm15218414)
Supplement: Supplementary file 1 [file jcm-15-08414-s001.zip › Supplementary Material.pdf]

Supplementary material

Title

Clinical phenotypes of oral and maxillofacial space infection: derivation, external assessment, and prognostic evaluation

Supplementary Methods

Methods S1. Phenotyping Variables and Clinical Outcomes

Twenty-four variables available at hospital presentation were used for phenotype derivation: age, sex, body mass index (BMI), temperature, heart rate, respiratory rate, mean arterial pressure (MAP), white blood cell count, platelet count, hemoglobin, absolute monocyte count, neutrophil-to-lymphocyte ratio (NLR), high-sensitivity C-reactive protein (hs-CRP), fibrinogen, potassium, sodium, chloride, calcium, total bilirubin, albumin, glucose, trismus, dysphagia, and dyspnea. BMI, MAP, and NLR were calculated from their component variables. Trismus was coded as absent, mild, moderate, or severe; sex, dysphagia, and dyspnea were coded as binary variables.

The traditional severity score (TSS) was calculated according to the fascial-space weighting system described by Flynn et al. [3,4]. Anatomical variables, TSS, treatment variables, and clinical outcomes were not included as clustering inputs and were analyzed in relation to the final phenotypes. The primary composite outcome included intensive care unit admission, tracheostomy, septic shock, or descending mediastinal infection. The secondary composite outcome included a hospital stay longer than 14 days, repeat debridement, or use of special-grade antibiotics.

Methods S2. Missing Data and Preprocessing

Missing continuous baseline values were imputed using multiple imputation by chained equations with predictive mean matching, yielding 20 completed datasets [15]. Anatomical space indicators were used as auxiliary predictors in the imputation models but were not included among the 24 clustering variables. BMI, MAP, and NLR were recalculated after imputation, and observed and imputed distributions were examined for plausibility. Right-skewed continuous variables were log-transformed. Continuous variables were standardized within each completed dataset, while categorical variables retained their prespecified coding. Spearman correlations among baseline clinical variables were summarized in a heatmap (Figure S2). The same preprocessing rules derived from the development cohort were then applied to the external validation cohort.

Methods S3. Phenotype Derivation and Selection

Ordering Points To Identify the Clustering Structure (OPTICS) reachability analysis was performed in each of the 20 imputed development datasets to explore the underlying density structure [16]. OPTICS used minPts = 10 and $\xi = 0.05$ (Figure S3). Consensus k-means clustering was then performed for candidate solutions from K = 2 to K = 6 [17,18].

Candidate solutions were evaluated using the consensus matrix, cumulative distribution function (CDF) of consensus values, change in CDF area, proportion of ambiguous clustering (PAC), cluster consensus, pairwise adjusted Rand index (ARI), silhouette width, phenotype size, and assignment confidence [16-20]. Assignment quality was further assessed using the negative silhouette fraction, low-confidence fraction, size imbalance ratio, within-cluster consensus, consensus margin, and minimum pairwise co-sampling. Low-confidence membership was defined as within-cluster consensus < 0.80 or consensus margin < 0.10.

Clustering stability and assignment quality were the main criteria for selecting K, with clinical interpretability as an additional consideration. No formal numerical weights were assigned, and investigators selected K before accessing outcome data.

Methods S4. Phenotype Characterization and Sensitivity Analysis

Phenotype characteristics were summarized on their original clinical scales. Standardized phenotype profiles were used to display clinical patterns, and mean pairwise absolute standardized differences were used to rank variables. Principal component analysis was used to display the multivariable phenotype structure in two dimensions. For descriptive visualization, alluvial plots showed the distribution of TSS categories (≤3, 4–9, and ≥10) across the derived phenotypes. TSS was modeled as a continuous variable in the prognostic models.

Latent class analysis (LCA) was performed as a sensitivity analysis using the same 24 variables separately in each of the 20 completed derivation datasets [21]. Continuous variables were categorized using tertile cutoffs derived from the development cohort, and the absent and mild trismus categories were combined. Candidate solutions were evaluated using the Bayesian information criterion (BIC), entropy, class size, clinical interpretability, and consistency across imputations. Posterior class-membership probabilities for the four-class LCA model are shown in Figure S6.

Methods S5. External Phenotype Assignment

The preprocessing rules and phenotype definitions derived from the development cohort were applied without change to the external validation cohort. Within each of the 20 imputed external datasets, patients were assigned to the nearest development-derived phenotype centroid using Euclidean distance. Final phenotype membership was determined by majority vote across imputations. Assignment confidence was defined as the highest vote count divided by 20; values below 0.80 were classified as low confidence. Ties were resolved by selecting the tied phenotype with the lowest mean distance.

Consensus k-means clustering was also performed independently in the external cohort for K = 2-6 as an exploratory analysis. Table S4 reports the clustering diagnostics. Mean pairwise ARI summarized agreement among partitions from the 20 imputed external datasets. Under this independent analysis, membership was classified as low confidence when within-cluster consensus was below 0.80 or the consensus margin was below 0.10. At the K value selected in the development cohort, ARI was used to compare the corresponding independent external partition with the frozen phenotype assignments. External outcome analyses used the frozen phenotype labels. Clinical profiles were compared between the development and external cohorts (Table S5; Figures S4 and S5).

Methods S6. Prognostic and Statistical Analyses

Three prespecified prognostic models were fitted for the primary composite outcome using Firth's mean bias-reduced logistic regression [22]: TSS alone, phenotype membership alone, and their combination. Each model was fitted once using the final phenotype assignments derived across the 20 imputed datasets. No imputation-specific outcome models were fitted, and predictions were not pooled across imputations. Phenotype was entered as a categorical variable, with Phenotype 1 as the reference category. Associations were reported as odds ratios (ORs) with 95% confidence intervals (CIs). Model performance was assessed using the area under the receiver operating characteristic curve (AUC), Brier score, calibration intercept, calibration slope, and decision-curve analysis [23-25].

Internal validation used 1,000 bootstrap resamples of the development cohort. In each resample, the outcome models were refitted using their original specifications and the final phenotype assignments. The refitted models were evaluated in both the bootstrap sample and the original development cohort. Optimism was calculated as the difference between these performance estimates and averaged across the 1,000 resamples. Mean optimism was then subtracted from each original model's apparent performance. Phenotype assignments remained fixed throughout; multiple imputation, consensus clustering, and K selection were not repeated. This analysis assessed the outcome models conditional on the derived phenotypes. Development-cohort performance estimates in Table 3 represent apparent performance, and bootstrap optimism-corrected AUCs are reported separately as point estimates.

For the original external validation, we applied the models fitted in the development cohort without refitting or recalibration. The observed-to-expected (O/E) ratio was calculated as the number of observed events divided by the sum of predicted probabilities. Net benefit in the external cohort was summarized at threshold probabilities of 10%, 20%, 30%, and 40%.

Original predicted probabilities from each frozen model were converted to log-odds for exploratory post hoc recalibration. Intercept-only recalibration included the original predicted log-odds as an offset with its

coefficient fixed at one and estimated an intercept adjustment. Intercept-and-slope recalibration used the predicted log-odds as the sole predictor and estimated both the intercept and slope. Both recalibration models were fitted by maximum likelihood using binomial logistic regression in the external cohort. Phenotype definitions and assignments were unchanged. Recalibrated AUC, Brier score, and O/E estimates were calculated in the same 51 patients and reported as apparent performance without confidence intervals.

Continuous variables were summarized as mean (standard deviation) or median (interquartile range), as appropriate, and categorical variables as counts and percentages. All statistical tests were two-sided, with $p < 0.05$ indicating nominal statistical significance. p-values for exploratory between-group comparisons were not adjusted for multiple comparisons. Analyses were performed using R version 4.5.2 (R Foundation for Statistical Computing, Vienna, Austria).

Methods S7. Targeted Descriptive Overlap and Center Comparisons

Following the original analyses, we conducted post hoc descriptive assessments using the fixed phenotype assignments in the development (n = 287) and external (n = 51) cohorts. Recorded diabetes history was summarized as the proportion of patients with diabetes history within each phenotype and the distribution of patients with recorded diabetes history across phenotypes. Wilson 95% confidence intervals were calculated for the within-phenotype proportions, with phenotype assignments held fixed.

Anatomical severity was summarized using continuous TSS values and the descriptive categories ⩽3, 4 – 9, and ⩾10. Selected clinical characteristics were compared across phenotypes within the TSS 4 – 9 category in the development cohort. This category was selected post hoc because it contained at least 10 patients from each phenotype. These comparisons did not involve matching on exact TSS values. Differences in variables used for clustering were interpreted descriptively.

Selected clinical characteristics and missing-data patterns were compared between centers using observed values before imputation, with variable-specific denominators. For continuous variables, signed standardized mean differences (SMDs) were calculated as the difference between cohort means (external minus development) divided by the square root of the average of their variances. Binary variables were compared using the corresponding standardized differences in proportions, while multicategory variables were compared using percentage-point differences. No additional model fitting, multiple imputation, or significance testing was performed.

Methods S8. Paired Incremental Prognostic Performance Analysis

To assess the incremental prognostic contribution of phenotype membership beyond anatomical severity, post hoc paired comparisons were performed between the combined model (TSS plus phenotype membership) and the TSS-only model. Differences in the area under the receiver operating characteristic curve (AUC) and Brier score were calculated as the combined model minus the TSS-only model.

In the development cohort, paired differences were estimated using bootstrap optimism-corrected performance, with 95% confidence intervals (CIs) derived from a two-stage paired bootstrap procedure. In the external cohort, the original frozen model predictions were compared without refitting or recalibration, and 95% CIs were estimated using paired patient-level bootstrap resampling.

Positive AUC differences and negative Brier score differences favored the combined model. Phenotype assignments were held fixed during resampling; multiple imputation, clustering, and selection of the number of phenotypes were not repeated. Therefore, the development CIs reflect uncertainty conditional on the derived phenotypes rather than the complete phenotype-development process. CIs including zero were not interpreted as evidence of model equivalence.

Methods S9. Exploratory Glucose-Exclusion Sensitivity Analysis

In a post hoc sensitivity analysis, we repeated K = 4 clustering after excluding glucose from the original 24-variable feature set. The remaining 23 variables, preprocessing procedures, and original 20 imputed

development datasets were retained. Multiple imputation was not repeated. Agreement between the original and glucose-excluded partitions was assessed using the adjusted Rand index (ARI). For each original phenotype, we identified the best-matched cluster in the glucose-excluded solution and calculated the Jaccard coefficient as the size of their intersection divided by the size of their union. This analysis assessed the sensitivity of the K = 4 solution to glucose exclusion.

Supplementary Tables

Table S1. Consensus clustering diagnostics for candidate solutions in the development cohort (n = 287).

Number of clusters	PAC[a]	CDF area	Δ area[b]	Mean cluster consensus[c]	Mean pairwise ARI[d]	Mean silhouette width
2	0.375	0.302	-	0.866	0.644	0.237
3	0.512	0.507	0.205	0.859	0.710	0.135
4	0.188	0.648	0.142	0.915	0.889	0.135
5	0.196	0.771	0.123	0.884	0.876	0.114
6	0.305	0.813	0.042	0.723	0.738	0.104

[a] PAC represents the proportion of patient-pair consensus values within the ambiguous range. Lower values indicate more stable separation.

[b] Δ area was calculated as CDF area(K) − CDF area(K − 1), representing the difference in CDF area between consecutive cluster solutions.

[c] Mean cluster consensus is the unweighted mean of the cluster-specific consensus values.

[d] Mean pairwise ARI summarizes agreement among phenotype assignments across the 20 imputed datasets after adjustment for chance.

Abbreviations: ARI, adjusted Rand index; CDF, cumulative distribution function; PAC, proportion of ambiguous clustering.

Table S2. Assignment quality and phenotype sizes for candidate clustering solutions in the development cohort (n = 287).

Number of clusters	Negative silhouette fraction[a]	Low-confidence fraction[b]	Phenotype size (min, max)	Size imbalance ratio[c]	Mean within-cluster consensus[d]	Mean consensus margin[e]	Minimum pairwise co-sampling[f]
2	4.7%	18.8%	35, 252	7.20	0.854	0.214	268
3	9.1%	59.9%	33, 158	4.79	0.848	0.195	270
4	4.5%	12.9%	31, 150	4.84	0.912	0.410	265
5	5.4%	17.8%	30, 82	2.73	0.875	0.369	272
6	10.8%	58.9%	14, 98	7.00	0.698	0.135	269

[a] The negative silhouette fraction is the proportion of patients with an individual silhouette width below zero.

[b] Membership was classified as low confidence when within-cluster consensus was below 0.80 or the consensus margin was below 0.10.

[c] The size imbalance ratio is the size of the largest phenotype divided by that of the smallest phenotype.

[d] Within-cluster consensus is the mean consensus between a patient and the other patients assigned to the same phenotype.

[e] The consensus margin is the within-cluster consensus minus the highest mean consensus with any alternative phenotype.

[f] Minimum pairwise co-sampling is the smallest number of consensus repetitions in which any patient pair was sampled together.

Table S3. Latent class analysis diagnostics for candidate solutions in the development cohort (n = 287).

Number of classes	Statistic		Class size n (%)					
	BIC	Entropy	1	2	3	4	5	6
2	13,791.944	0.929	98 (34.1%)	189 (65.9%)				
3	13,885.136	0.913	101 (35.2%)	91 (31.7%)	95 (33.1%)			
4	13,783.324	0.936	103 (35.9%)	39 (13.6%)	84 (29.3%)	61 (21.3%)		
5	14,006.868	0.934	79 (27.5%)	38 (13.2%)	41 (14.3%)	81 (28.2%)	48 (16.7%)	
6	14,140.736	0.944	69 (24.0%)	39 (13.6%)	20 (7.0%)	47 (16.4%)	79 (27.5%)	33 (11.5%)

Latent class analysis used the same 24 presentation variables as the consensus clustering analysis. Continuous variables were categorized using development-derived tertile cutoffs, and the absent and mild trismus categories were combined before model fitting. Lower BIC values indicate a more favorable balance between model fit and complexity; entropy values closer to 1 indicate greater classification certainty. BIC and entropy are reported as mean values across the 20 imputed development datasets.

Abbreviation: BIC, Bayesian information criterion.

Table S4. Exploratory independent consensus clustering diagnostics in the external cohort (n = 51).

Number of clusters	PAC[a]	Mean pairwise ARI[b]	Mean silhouette width	Phenotype size (min, max)[c]	Mean within-cluster consensus[d]	Low-confidence fraction[e]	Mean consensus margin[f]
2	0.484	0.538	0.233	15, 36	0.873	0.275	0.325
3	0.405	0.737	0.174	6, 33	0.855	0.373	0.326
4	0.324	0.754	0.237	4, 25	0.815	0.294	0.278
5	0.467	0.703	0.174	1, 31	0.654	0.882	0.307
6	0.407	0.571	0.136	1, 18	0.674	0.804	0.248

[a] Independent consensus k-means clustering was performed within the external cohort for K = 2–6. Frozen K = 4 assignments were used for external outcome analyses.

[b] Mean pairwise ARI summarizes agreement among partitions derived from the 20 imputed external datasets.

[c] Cluster-size ranges refer to the pooled independent cluster labels and differ from the group sizes obtained by frozen assignment.

[d] Within-cluster consensus is the mean consensus between a patient and the other patients assigned to the same phenotype.

[e] Low-confidence membership in the independent clustering analysis was defined as within-cluster consensus <0.80 or consensus margin <0.10. This differs from the vote-based confidence used for frozen assignment.

[f] The consensus margin is the within-cluster consensus minus the highest mean consensus with any alternative phenotype.

Abbreviations: ARI, adjusted Rand index; PAC, proportion of ambiguous clustering.

Table S5. Presentation characteristics of the four OMSI phenotypes in the external validation cohort.

Characteristic	Total	Phenotype			
		1	2	3	4
No.	51	25	6	16	4
Demographic variables					
Male sex, n (%)	29 (56.9%)	19 (76.0%)	3 (50.0%)	6 (37.5%)	1 (25.0%)
Age, years, median (IQR)	60.0 (50.5, 73.0)	60.0 (52.0, 74.0)	69.5 (57.8, 72.2)	53.5 (35.8, 58.8)	70.5 (66.0, 76.2)
BMI, kg/m², median (IQR)	23.2 (20.2, 26.2)	23.9 (20.4, 27.1)	22.6 (20.7, 24.7)	21.3 (18.9, 24.7)	26.8 (25.6, 27.9)
Vital signs					
Temperature, °C, median (IQR)	36.4 (36.2, 36.6)	36.5 (36.3, 36.6)	38.4 (38.1, 38.5)	36.3 (36.2, 36.4)	36.2 (36.2, 36.3)
Heart rate, beats/min, median (IQR)	84 (75, 96)	85 (72, 96)	94 (88, 106)	81 (74, 86)	85 (82, 90)
Respiratory rate, breaths/min, median (IQR)	20 (19, 20)	20 (19, 20)	24 (20, 26)	20 (19, 20)	20 (20, 22)
Mean arterial pressure, mmHg, median (IQR)	93.3 (84.8, 101.7)	92.7 (86.0, 101.3)	96.7 (91.7, 106.2)	92.7 (77.6, 95.6)	109.3 (107.0, 113.1)
Hematological measures					
White blood cell count, ×10⁹/L, median (IQR)	12.80 (10.07, 15.64)	14.37 (11.74, 15.93)	12.82 (11.49, 17.77)	8.62 (6.88, 10.38)	14.91 (13.56, 17.09)
Platelet count, ×10⁹/L, median (IQR)	236 (182, 302)	215 (174, 302)	208 (187, 216)	259 (207, 307)	278 (212, 330)
Hemoglobin, g/L, mean (SD)	137.3 (22.0)	138.9 (26.1)	126.3 (16.2)	136.9 (18.1)	145.3 (15.4)
Absolute monocyte count, ×10⁹/L, median (IQR)	0.89 (0.61, 1.19)	1.17 (0.98, 1.28)	0.63 (0.29, 1.45)	0.61 (0.47, 0.85)	0.88 (0.81, 0.99)
Inflammatory and coagulation markers					
NLR, median (IQR)	7.21 (4.70, 12.62)	7.79 (5.29, 13.00)	13.35 (8.25, 16.46)	5.51 (1.92, 6.84)	13.31 (11.05, 15.06)
hs-CRP, mg/L, median (IQR)	84.2 (37.0, 143.8)	97.3 (50.5, 139.9)	225.2 (153.6, 269.3)	25.2 (6.0, 66.1)	149.6 (103.2, 217.6)
Fibrinogen, g/L, median (IQR)	4.99 (4.46, 6.13)	5.29 (4.45, 6.50)	5.58 (5.00, 6.55)	4.69 (4.19, 5.04)	5.82 (5.29, 6.09)
Serum biochemical measures					
K, mmol/L, median (IQR)	3.88 (3.56, 4.10)	3.83 (3.49, 4.01)	3.88 (3.45, 4.18)	3.95 (3.76, 4.14)	3.94 (3.79, 4.02)
Na, mmol/L, median (IQR)	138.7 (136.4, 141.1)	137.6 (135.4, 140.2)	137.8 (135.4, 138.5)	141.2 (139.5, 142.8)	135.9 (134.7, 136.9)
Cl, mmol/L, median (IQR)	102.0 (98.9,	101.0 (98.3,	103.2 (97.5,	104.1 (102.0,	98.3 (97.2,

	104.8)	103.4)	104.0)	105.7)	100.0)
Ca, mmol/L, mean (SD)	2.18 (0.13)	2.14 (0.16)	2.17 (0.08)	2.24 (0.07)	2.21 (0.16)
Total bilirubin, μmol/L, median (IQR)	13.4 (9.3, 23.4)	16.0 (10.6, 23.9)	23.9 (14.0, 31.3)	10.1 (8.2, 12.4)	21.6 (9.3, 38.0)
Albumin, g/L, mean (SD)	38.8 (5.8)	38.1 (6.2)	36.8 (6.9)	40.5 (5.2)	39.9 (2.8)
Glucose, mmol/L, median (IQR)	6.1 (5.1, 7.3)	6.1 (5.0, 7.4)	6.4 (6.0, 6.9)	5.6 (4.8, 6.2)	16.7 (14.9, 19.0)
Presenting symptoms					
Trismus, n (%)					
Absent	10 (19.6%)	5 (20.0%)	1 (16.7%)	4 (25.0%)	0 (0.0%)
Mild	9 (17.6%)	3 (12.0%)	0 (0.0%)	5 (31.2%)	1 (25.0%)
Moderate	14 (27.5%)	5 (20.0%)	2 (33.3%)	5 (31.2%)	2 (50.0%)
Severe	18 (35.3%)	12 (48.0%)	3 (50.0%)	2 (12.5%)	1 (25.0%)
Dysphagia, n (%)	6 (11.8%)	3 (12.0%)	1 (16.7%)	1 (6.2%)	1 (25.0%)
Dyspnea, n (%)	9 (17.6%)	6 (24.0%)	1 (16.7%)	1 (6.2%)	1 (25.0%)
Clinical history					
Recorded diabetes history, n (%)	15 (29.4%)	8 (32.0%)	1 (16.7%)	2 (12.5%)	4 (100.0%)

Recorded diabetes history was not included among the 24 variables used for phenotype derivation.

Abbreviations: BMI, body mass index; hs-CRP, high-sensitivity C-reactive protein; IQR, interquartile range; NLR, neutrophil-to-lymphocyte ratio; OMSI, oral and maxillofacial space infection; SD, standard deviation.

Table S6. Associations of phenotype membership and TSS with the primary composite outcome in the development cohort.

Model	Predictor	β	OR (95% CI)	*p*
Phenotype model	Phenotype 1	Reference	1.00 (reference)	-
	Phenotype 2 vs Phenotype 1	0.307	1.36 (0.54, 3.42)	0.516
	Phenotype 3 vs Phenotype 1	−2.209	0.11 (0.02, 0.59)	0.010
	Phenotype 4 vs Phenotype 1	−0.456	0.63 (0.19, 2.12)	0.460
TSS model	TSS, per 1-point increase	0.261	1.30 (1.19, 1.42)	<0.001
Phenotype + TSS model	TSS, per 1-point increase	0.233	1.26 (1.15, 1.38)	<0.001
	Phenotype 1	Reference	1.00 (reference)	-
	Phenotype 2 vs Phenotype 1	0.057	1.06 (0.39, 2.89)	0.911
	Phenotype 3 vs Phenotype 1	−1.282	0.28 (0.05, 1.59)	0.150
	Phenotype 4 vs Phenotype 1	−0.397	0.67 (0.19, 2.38)	0.538

Abbreviations: CI, confidence interval; OR, odds ratio; β, regression coefficient; TSS, traditional severity score.

Table S7. Primary composite outcome by phenotype in the development and external validation cohorts.

Cohort	Phenotype	n	Primary composite outcome, n (%)
Development	Phenotype 1	150	24 (16.0%)

	Phenotype 2	35	7 (20.0%)
	Phenotype 3	71	1 (1.4%)
	Phenotype 4	31	3 (9.7%)
External validation	Phenotype 1	25	9 (36.0%)
	Phenotype 2	6	6 (100.0%)
	Phenotype 3	16	4 (25.0%)
	Phenotype 4	4	3 (75.0%)

Table S8. External validation performance and decision-curve net benefit of the TSS, phenotype, and combined models.

Model	AUC (95% CI)	Brier score	Calibration intercept	Calibration slope	O/E ratio	Net benefit at 10%[a]	Net benefit at 20%[a]	Net benefit at 30%[a]	Net benefit at 40%[a]
TSS model	0.926 (0.850, 0.981)	0.301	2.537	2.282	4.425	0.253	0.157	0.078	0.059
Phenotype model	0.665 (0.513, 0.796)	0.328	1.926	0.549	3.620	0.316	0.118	0.000	0.000
Combined model	0.891 (0.785, 0.971)	0.303	2.531	1.464	4.424	0.307	0.118	0.059	0.039

[a] Net benefit was evaluated at threshold probabilities of 10%, 20%, 30%, and 40%.

Abbreviations: AUC, area under the receiver operating characteristic curve; CI, confidence interval; O/E, observed-to-expected ratio; TSS, traditional severity score.

Table S9. Exploratory recalibration of the frozen models in the external cohort (51 patients, 22 events).

Model	Updating approach	AUC	Brier score	O/E	Updating intercept (a)	Updating slope (b)
TSS	Original frozen model	0.926	0.301	4.425	-	-
TSS	Intercept only	0.926	0.129	1.000	2.537	1.000
TSS	Intercept and slope	0.926	0.114	1.000	6.208	2.282
Phenotype	Original frozen model	0.665	0.328	3.620	-	-
Phenotype	Intercept only	0.665	0.235	1.000	1.926	1.000
Phenotype	Intercept and slope	0.665	0.228	1.000	0.972	0.549
Combined	Original frozen model	0.891	0.303	4.424	-	-
Combined	Intercept only	0.891	0.144	1.000	2.531	1.000
Combined	Intercept and slope	0.891	0.138	1.000	3.765	1.464

Abbreviations: AUC, area under the receiver operating characteristic curve; O/E, observed-to-expected ratio; TSS, traditional severity score.

Table S10. Overlap of candidate phenotypes with recorded diabetes and anatomical severity.

(a) Recorded diabetes within and across phenotypes

Cohort	Phenotype	Diabetes / phenotype	Wilson 95% CI	Phenotype / all diabetes
Development	P1	21/150 (14.0%)	9.3–20.5%	21/64 (32.8%)
Development	P2	3/35 (8.6%)	3.0–22.4%	3/64 (4.7%)
Development	P3	11/71 (15.5%)	8.9–25.7%	11/64 (17.2%)
Development	P4	29/31 (93.5%)	79.3–98.2%	29/64 (45.3%)
External	P1	8/25 (32.0%)	17.2–51.6%	8/15 (53.3%)
External	P2	1/6 (16.7%)	3.0–56.4%	1/15 (6.7%)
External	P3	2/16 (12.5%)	3.5–36.0%	2/15 (13.3%)
External	P4	4/4 (100.0%)	51.0–100.0%	4/15 (26.7%)

(b) Anatomical severity across fixed phenotype assignments

Cohort	Phenotype	n	Median TSS	TSS ≤3, n (%)	TSS 4–9, n (%)	TSS ≥10, n (%)
Development	P1	150	9	8 (5.3%)	76 (50.7%)	66 (44.0%)
Development	P2	35	11	2 (5.7%)	10 (28.6%)	23 (65.7%)
Development	P3	71	5	25 (35.2%)	38 (53.5%)	8 (11.3%)
Development	P4	31	9	1 (3.2%)	16 (51.6%)	14 (45.2%)
External	P1	25	6	2 (8.0%)	17 (68.0%)	6 (24.0%)
External	P2	6	10.5	0 (0.0%)	2 (33.3%)	4 (66.7%)
External	P3	16	3	8 (50.0%)	6 (37.5%)	2 (12.5%)
External	P4	4	—	0 (0.0%)	2 (50.0%)	2 (50.0%)

(c) Clinical presentation within the TSS 4–9 category in the development cohort

Phenotype	n	Median TSS	Temperature (°C)	Glucose (mmol/L)	hs-CRP (mg/L)	NLR
P1	76	7	36.60 (n=76)	6.00 (n=71)	98.10 (n=71)	9.00 (n=76)
P2	10	7	38.50 (n=10)	5.48 (n=10)	94.60 (n=10)	8.09 (n=10)
P3	38	7	36.45 (n=38)	4.98 (n=35)	23.90 (n=35)	3.62 (n=38)
P4	16	7	36.50 (n=16)	14.27 (n=13)	85.00 (n=13)	11.57 (n=16)

Recorded diabetes history was not included among the 24 clustering variables. In (a), within-phenotype diabetes prevalence and the distribution of patients with diabetes across phenotypes use different denominators. Wilson 95% confidence intervals (CIs) apply to within-phenotype prevalence, conditional on fixed phenotype assignments. In (b), TSS was categorized as ⩽3, 4 – 9, and ⩾10, with percentages calculated within each phenotype. Median TSS was not reported for external Phenotype 4 (n = 4). In (c), continuous variables are presented as observed-data medians, with variable-specific sample sizes in parentheses. The TSS 4–9 subgroup analysis was conducted post hoc without exact TSS matching.

Abbreviations: CI, confidence interval; hs-CRP, high-sensitivity C-reactive protein; NLR, neutrophil-to-lymphocyte ratio; TSS, traditional severity score.

Table S11. Selected between-center differences in clinical characteristics and data completeness.

Category	Characteristic	Development (n=287)	External (n=51)	Difference
Clinical characteristics	TSS, points	8.0 [5.0, 11.0]	6.0 [4.0, 10.0]	−0.330

Clinical characteristics	Heart rate, beats/min	93.0 [81.0, 105.0]	84.0 [75.0, 96.0]	−0.500
Clinical characteristics	Respiratory rate, breaths/min	18.0 [18.0, 20.0]	20.0 [19.0, 20.0]	+0.626
Clinical characteristics	Severe trismus, n (%)	203/287 (70.7%)	18/51 (35.3%)	−35.4 pp
Clinical characteristics	Diabetes history, n (%)	64/287 (22.3%)	15/51 (29.4%)	+0.163
Clinical characteristics	Odontogenic source, n (%)	254/287 (88.5%)	37/51 (72.5%)	−16.0 pp
Data completeness	Missing BMI, n (%)	1/287 (0.3%)	16/51 (31.4%)	+31.0 pp
Data completeness	Missing glucose, n (%)	25/287 (8.7%)	14/51 (27.5%)	+18.7 pp
Data completeness	Missing hs-CRP, n (%)	19/287 (6.6%)	14/51 (27.5%)	+20.8 pp
Data completeness	Missing fibrinogen, n (%)	9/287 (3.1%)	14/51 (27.5%)	+24.3 pp
Data completeness	Missing albumin, n (%)	26/287 (9.1%)	14/51 (27.5%)	+18.4 pp

Continuous variables are presented as median [interquartile range], and categorical variables as counts and percentages, using observed data before imputation. Differences (external minus development) are expressed as signed standardized mean differences (SMDs) for TSS, heart rate, respiratory rate, and recorded diabetes history, and as percentage-point differences (pp) for severe trismus, odontogenic source, and missing-data proportions. Continuous-variable SMDs were calculated using means and standard deviations, although medians and interquartile ranges are displayed.

Abbreviations: BMI, body mass index; hs-CRP, high-sensitivity C-reactive protein; ICU, intensive care unit; IQR, interquartile range; pp, percentage points; SMD, standardized mean difference; TSS, traditional severity score.

Table S12. Paired differences in prognostic performance between the combined and TSS-only models.

Cohort	Metric	Combined − TSS	95% CI	Method
Development	AUC	+0.0015	−0.0144 to +0.0498	Conditional two-stage bootstrap
Development	Brier score	+0.0025	−0.0035 to +0.0037	Conditional two-stage bootstrap
External	AUC	−0.0345	−0.0886 to +0.0042	Paired frozen predictions
External	Brier score	+0.0028	−0.0113 to +0.0219	Paired frozen predictions

Differences were calculated as combined-model (TSS plus phenotype membership) minus TSS-only model performance. Positive AUC differences and negative Brier score differences favor the combined model. Development estimates were optimism-corrected with phenotype assignments held fixed, whereas external estimates used the original frozen predictions without refitting or recalibration. Unlike the apparent development Brier scores in manuscript Table 3, the development Brier score differences reported here were based on optimism-corrected estimates.

Abbreviations: AUC, area under the receiver operating characteristic curve; CI, confidence interval; TSS, traditional severity score.

Table S13. Sensitivity of the original four-group partition to exclusion of glucose.

Original phenotype	Original n	Matched new cluster n	Intersection / union	Best-match Jaccard
P1	150	97	76/171	0.444
P2	35	42	35/42	0.833
P3	71	62	59/74	0.797
P4	31	86	16/101	0.158

The adjusted Rand index (ARI) between the original and glucose-excluded K = 4 partitions was 0.4298. Patients in the original Phenotype 4 were redistributed across the four new clusters (16, 4, 9, and 2 patients). Best-match Jaccard coefficients were calculated as the intersection divided by the union of matched groups. The analysis reused the original 20 imputed datasets without repeating imputation.

Abbreviations: ARI, adjusted Rand index.

Supplementary Figures

Figure S1. Patient selection flow diagram.

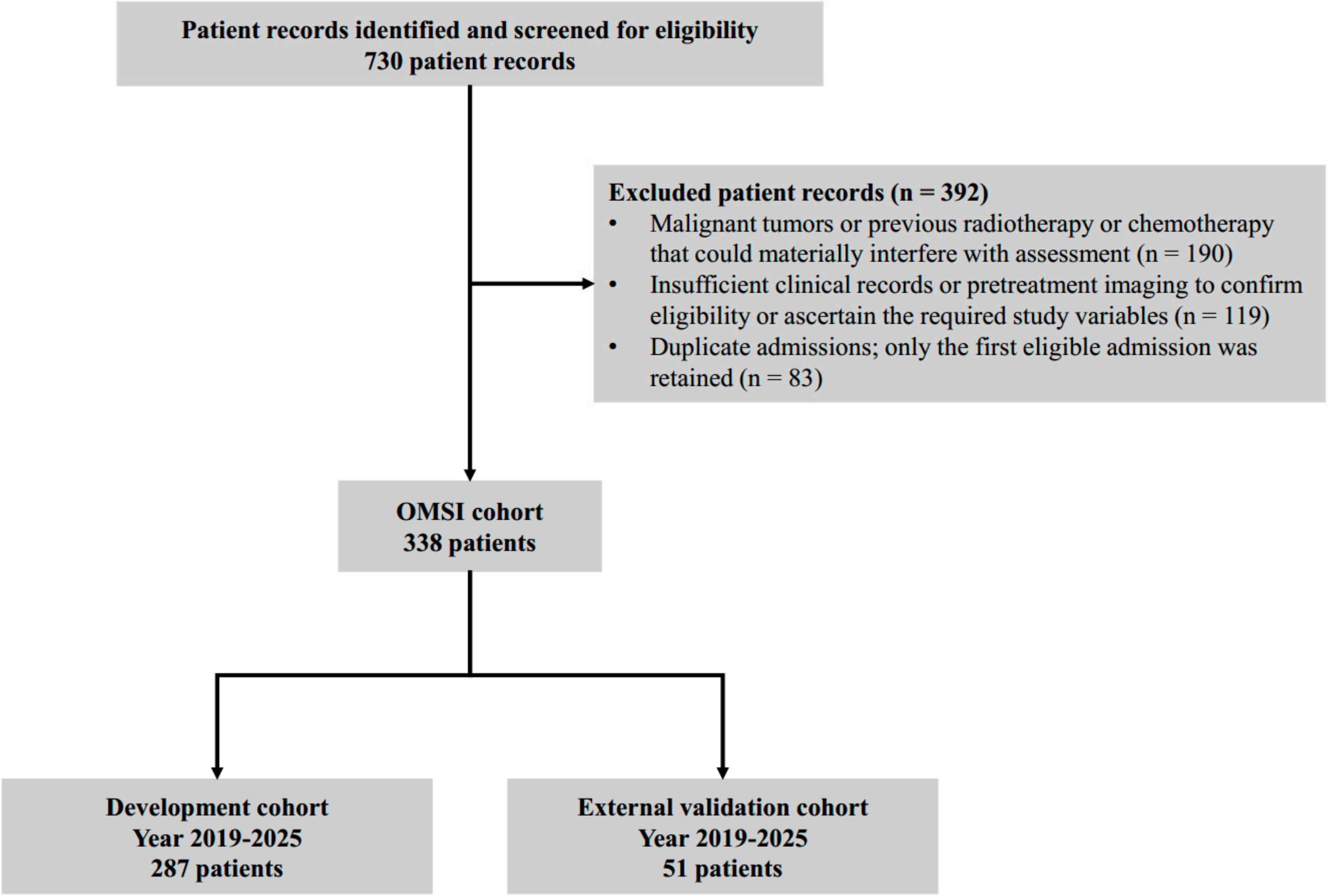

Figure S2. Correlation heatmap of baseline clinical variables assessed during preprocessing.

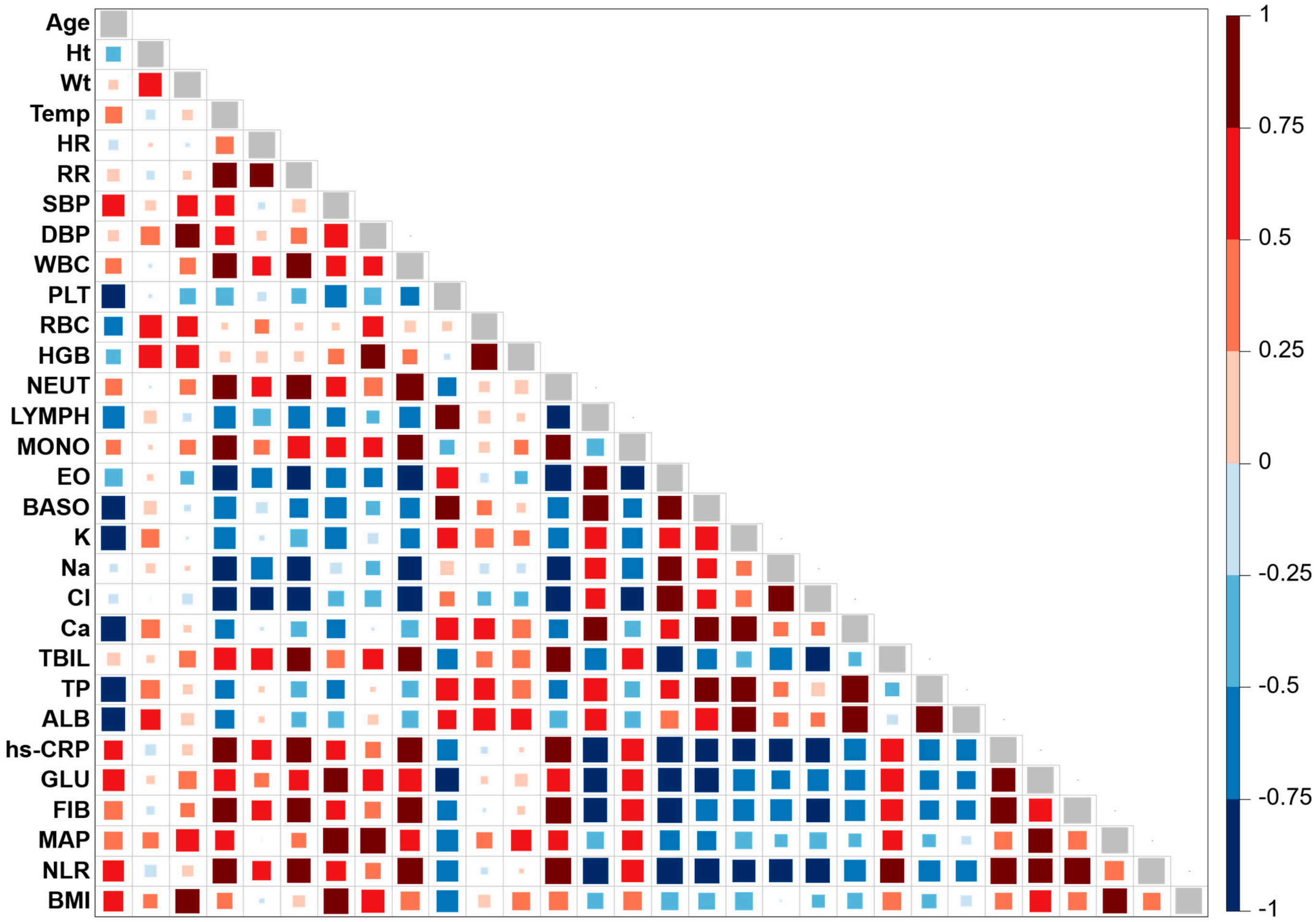

Spearman correlation coefficients among baseline clinical variables assessed during preprocessing are shown. Darker red and blue indicate stronger positive and negative correlations, respectively.

Abbreviations: ALB, albumin; BASO, absolute basophil count; BMI, body mass index; Ca, calcium; Cl, chloride; DBP, diastolic blood pressure; EO, absolute eosinophil count; FIB, fibrinogen; GLU, glucose; HGB, hemoglobin; HR, heart rate; hs-CRP, high-sensitivity C-reactive protein; Ht, height; K, potassium; LYMPH, absolute lymphocyte count; MAP, mean arterial pressure; MONO, absolute monocyte count; Na, sodium; NEUT, absolute neutrophil count; NLR, neutrophil-to-lymphocyte ratio; PLT, platelet count; RBC, red blood cell count; RR, respiratory rate; SBP, systolic blood pressure; TBIL, total bilirubin; Temp, temperature; TP, total protein; WBC, white blood cell count; Wt, weight.

Figure S3. OPTICS reachability plots across 20 multiply imputed datasets.

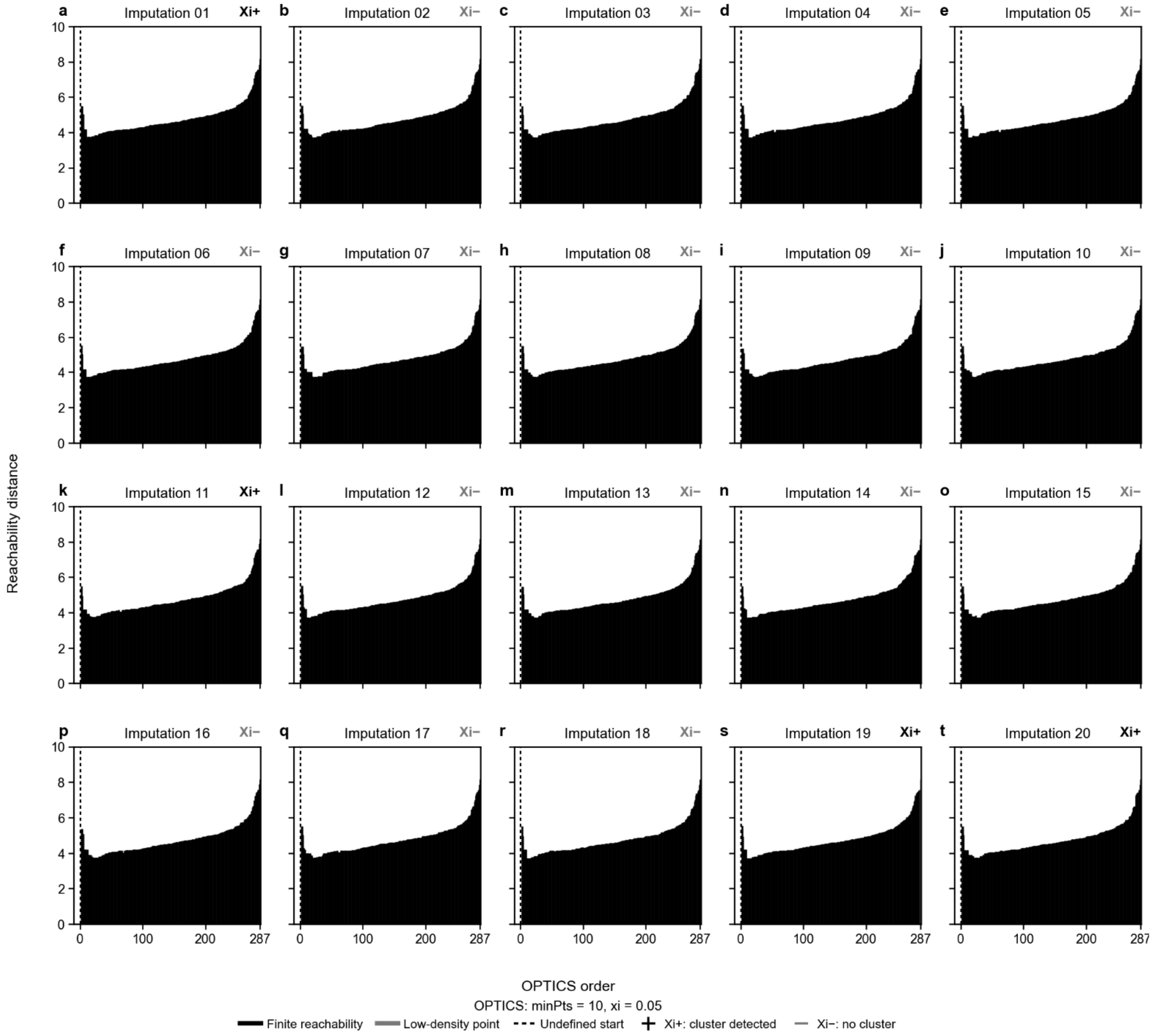

The reachability plots were used as an exploratory assessment of density structure before consensus k-means clustering.

Figure S4. Cross-cohort comparison of phenotype profiles: age through hemoglobin.

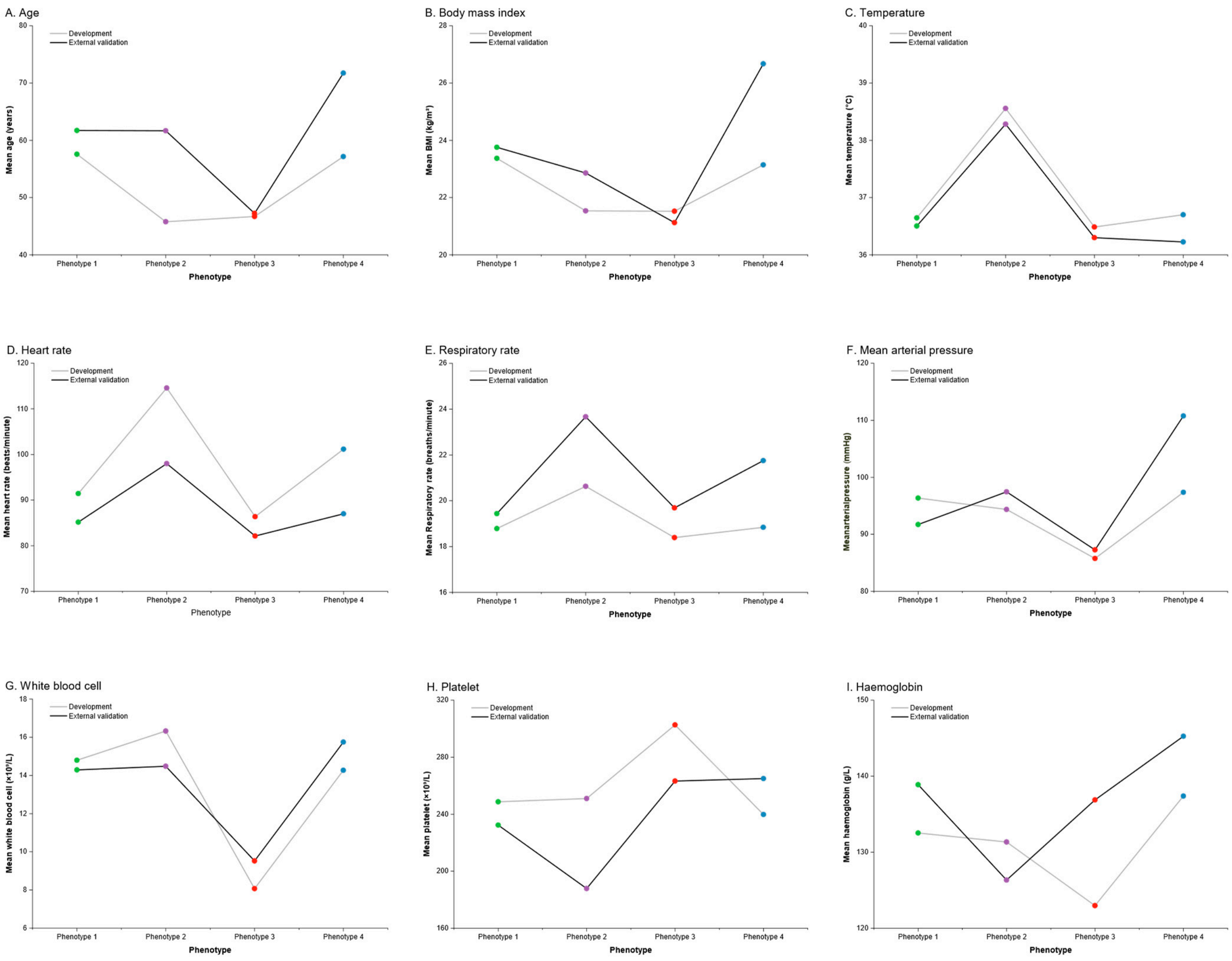

Each panel compares mean values of a continuous variable across the four phenotypes in the development (n = 287) and external validation (n = 51) cohorts.

Figure S5. Cross-cohort comparison of phenotype profiles: absolute monocyte count through glucose.

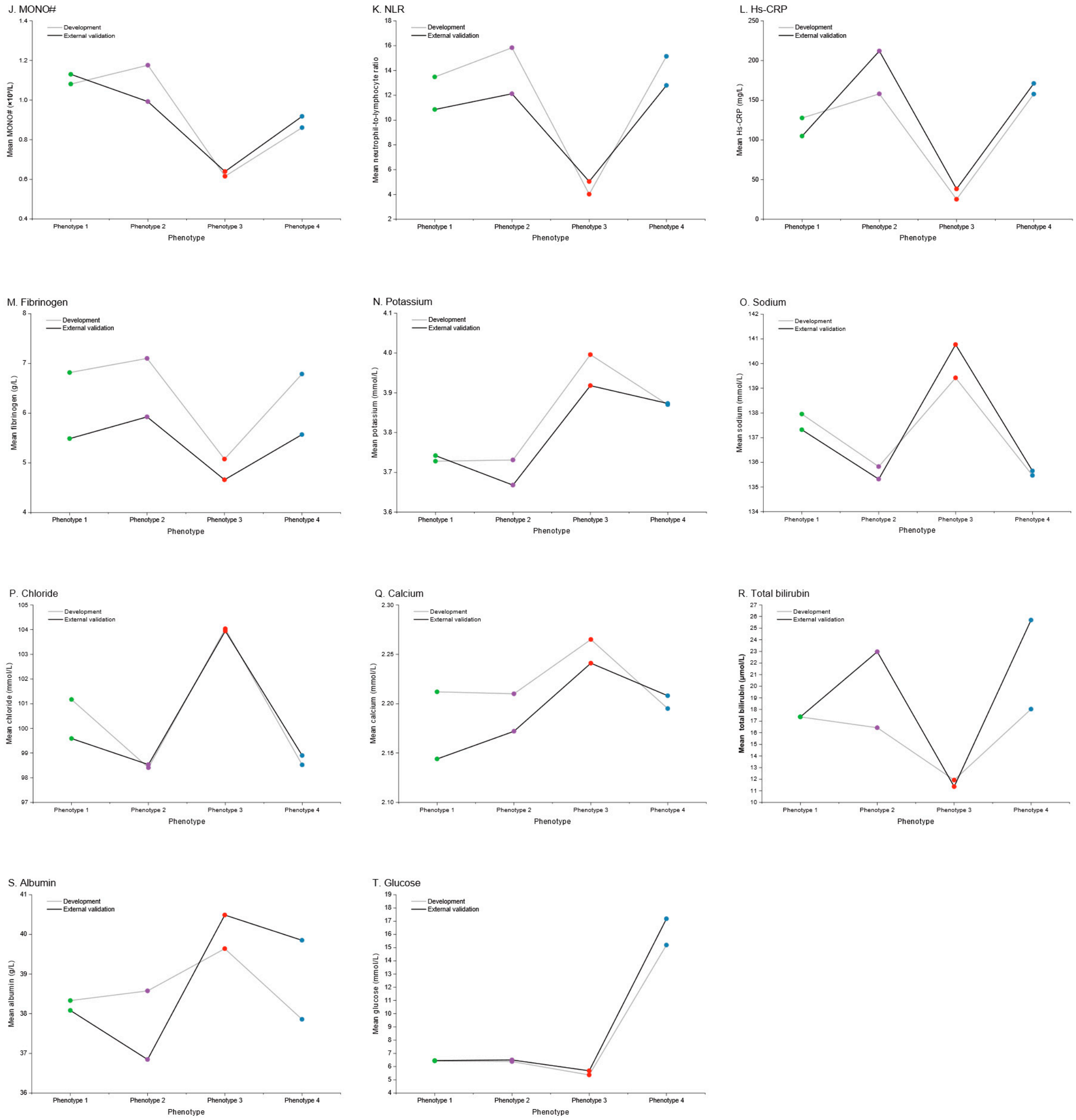

Each panel compares mean values of a continuous variable across the four phenotypes in the development (n = 287) and external validation (n = 51) cohorts.

Figure S6. Posterior class-membership probabilities for the four-class latent class model in the development cohort (n = 287).

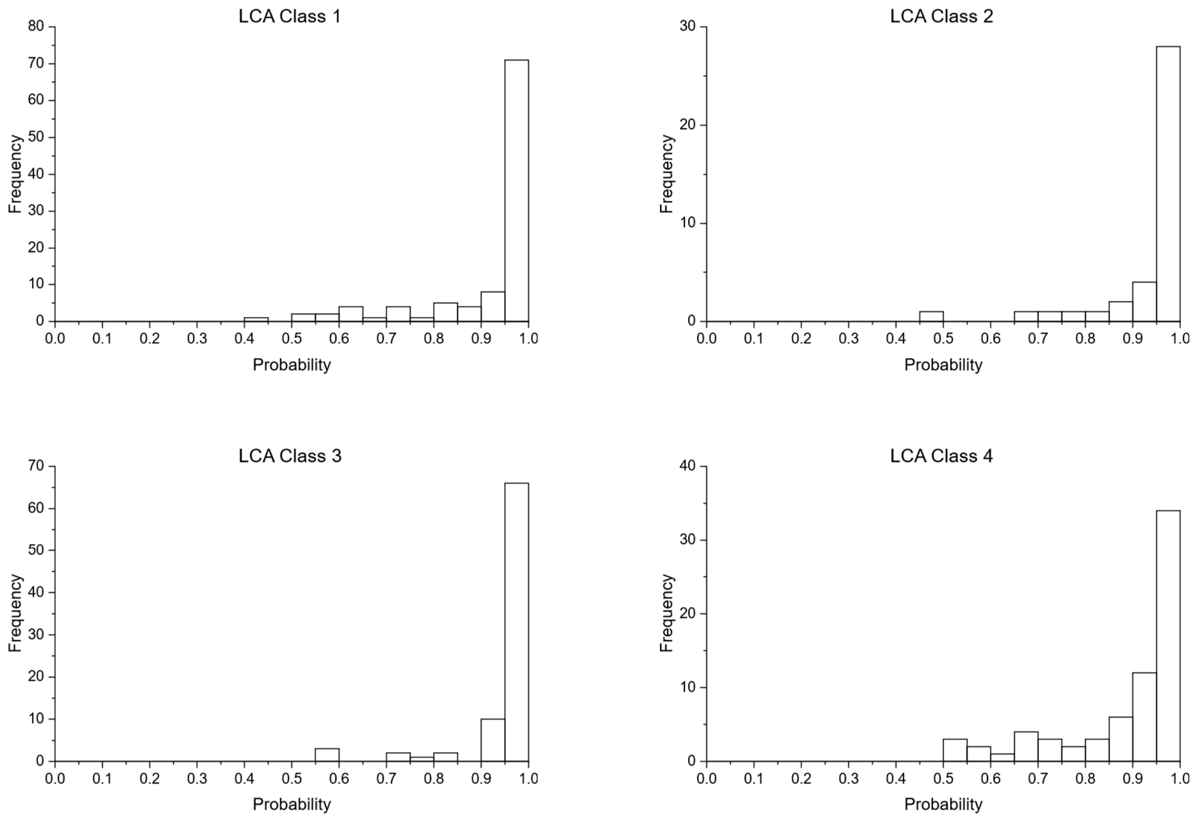

Histograms show posterior class-membership probabilities for the four-class LCA model. Higher probabilities indicate greater certainty of class assignment.
